# *In Vivo* Differences between Two Optical Isomers of Radioiodinated *o*-iodo-*trans*-decalinvesamicol for Use as a Radioligand for the Vesicular Acetylcholine Transporter

**DOI:** 10.1371/journal.pone.0146719

**Published:** 2016-01-11

**Authors:** Izumi Uno, Takashi Kozaka, Daisuke Miwa, Yoji Kitamura, Mohammad Anwar-ul Azim, Kazuma Ogawa, Junichi Taki, Seigo Kinuya, Kazuhiro Shiba

**Affiliations:** 1 Division of Tracer Kinetics, Advanced Science Research Center, Kanazawa University, Ishikawa, 920–8640, Japan; 2 Institute of Medical, Pharmaceutical and Health Sciences, Kanazawa University, Ishikawa, 920–8640, Japan; 3 Clinical Laboratory, Kanazawa University Hospital, Kanazawa, Ishikawa, 920–8640, Japan; 4 National Institute of Nuclear Medicine and Allied Sciences; Bangladesh Atomic Energy Commission, BSM Medical University Campus, Dhaka-1000, Bangladesh; University of São Paulo, BRAZIL

## Abstract

**Purpose:**

To develop a superior VAChT imaging probe for SPECT, radiolabeled (-)-OIDV and (+)-OIDV were isolated and investigated for differences in their binding affinity and selectivity to VAChT, as well as their *in vivo* activities.

**Procedures:**

Radioiodinated *o*-iodo-*trans*-decalinvesamicol ([^125^I]OIDV) has a high binding affinity for vesicular acetylcholine transporter (VAChT) both *in vitro* and *in vivo*. Racemic [^125^I]OIDV was separated into its two optical isomers (-)-[^125^I]OIDV and (+)-[^125^I]OIDV by HPLC. To investigate VAChT binding affinity (Ki) of two OIDV isomers, *in vitro* binding assays were performed. *In vivo* biodistribution study of each [^125^I]OIDV isomer in blood, brain regions and major organs of rats was performed at 2,30 and 60 min post-injection. *In vivo* blocking study were performed to reveal the binding selectivity of two [^125^I]OIDV isomers to VAChT *in vivo*. *Ex vivo* autoradiography were performed to reveal the regional brain distribution of two [^125^I]OIDV isomers and (-)-[^123^I]OIDV for SPECT at 60 min postinjection.

**Results:**

VAChT binding affinity (Ki) of (-)-[^125^I]OIDV and (+)-[^125^I]OIDV was 22.1 nM and 79.0 nM, respectively. At 2 min post-injection, accumulation of (-)-[^125^I]OIDV was the same as that of (+)-[^125^I]OIDV. However, (+)-[^125^I]OIDV clearance from the brain was faster than (-)-[^125^I]OIDV. At 30 min post-injection, accumulation of (-)-[^125^I]OIDV (0.62 ± 0.10%ID/g) was higher than (+)-[^125^I]OIDV (0.46 ± 0.07%ID/g) in the cortex. Inhibition of OIDV binding showed that (-)-[^125^I]OIDV was selectively accumulated in regions known to express VAChT in the rat brain, and *ex vivo* autoradiography further confirmed these results showing similar accumulation of (-)-[^125^I]OIDV in these regions. Furthermore, (-)-[^123^I]OIDV for SPECT showed the same regional brain distribution as (-)-[^125^I]OIDV.

**Conclusion:**

These results suggest that radioiodinated (-)-OIDV may be a potentially useful tool for studying presynaptic cholinergic neurons in the brain.

## Introduction

Alzheimer’s disease (AD) is a neurodegenerative disorder characterized by progressive reduction in cognitive function and memory, and is associated with amyloid-β[1,2] and tau protein deposits[3,4] and the dysfunction of cholinergic neurons and synapses[5–10]. Hence, visualization of any changes in cholinergic neurotransmission as well as amyloid accumulation in the brain is important for more accurate diagnosis of AD. Many amyloid imaging clinical trials, using compounds such as ^11^C-Pittsburgh Compound B (PIB)[11–13], are currently being performed. While there have been reports on the usefulness of amyloid imaging for earlier diagnosis of AD, other reports have shown no significant association between PIB accumulation and pathological amyloid density in the brain and the severity of dementia in AD[14–19]. Evaluation of the therapeutic efficacy of AD treatment will require future development of suitable imaging agents. The cholinergic system is thought to be highly associated with cognition, memory, and learning. At present, acetylcholine esterase inhibitors are commonly used for the treatment of cognitive dysfunction in AD patients. Presynaptic cholinergic function such as loss of choline acetyl transferase (ChAT) remarkably changed in AD[5,6]. Imaging of the presynaptic cholinergic function for diagnosis of AD is still an interesting research field in nuclear medicine. Among these cholinergic neuronal parameters, vesicular acetylcholine transporter (VAChT)[8–10] has been considered a cholinergic neuron terminals marker. In the central nervous system (CNS), VAChT is involved in the transportation of acetylcholine (ACh) in the synaptic vesicles. VAChT deficiency leads to lack of release of Ach to synaptic cleft in the brain, and alters cognitive functions such as social recognition, learning, and memory. Because synapse loss and dysfunction occur before fibrillary tau tangles emerge in the tauopathy mouse AD model[20], VAChT may be used as a possible *in vivo* target for diagnoses of AD.

It has been shown that vesamicol (2-(4-phenylpiperidino) cyclohexanol) binds to the ACh transporter on presynaptic acetylcholine storage vesicles and inhibits ACh uptake into the vesicle[21,22]. Many vesamicol analogs have been studied as putative VAChT imaging agents for use in the diagnosis of AD[23–29]. However, none of these analogues, as well as vesamicol itself, were shown to be suitable to proceed to clinical trials due to specific binding to σ receptors (σ-1, σ-2)[30]. In a recent report, we synthesized 2-Hydroxy-3-(4-(*o*-Iodo-phenylpiperidino))-*trans*-decalinvesamicol (OIDV), a new vesamicol analog with the framework of decalinvesamicol (DV)[31] and with radioiodine at the *ortho*-position of the 4-phenylpiperidine moiety. We demonstrated the high affinity and selectivity of the radioiodinated OIDV for VAChT both *in vitro* and *in vivo*[32,33]. In many cases, it is not uncommon for the optical isomers of a neuroreceptor agonist or antagonist to differ in their affinities and activities. As such, several radiolabeled vesamicol analogs developed for VAChT imaging have been shown to have one optical isomer having a higher affinity for VAChT than the other[31,34,35]. Therefore, to develop a superior VAChT imaging probe for SPECT, radiolabeled (-)-OIDV and (+)-OIDV were isolated and investigated for differences in their binding affinity and selectivity to VAChT, as well as their *in vivo* activities.

## Materials and Methods

### General

(+/-)-Vesamicol, (+)-pentazocine and DTG were purchased from Sigma-Aldrich Co. (St. Louis, MO). Radioisotopes were purchased from PerkinElmer, Inc. (Waltham, MA), unless otherwise noted.

**Fig 1:**
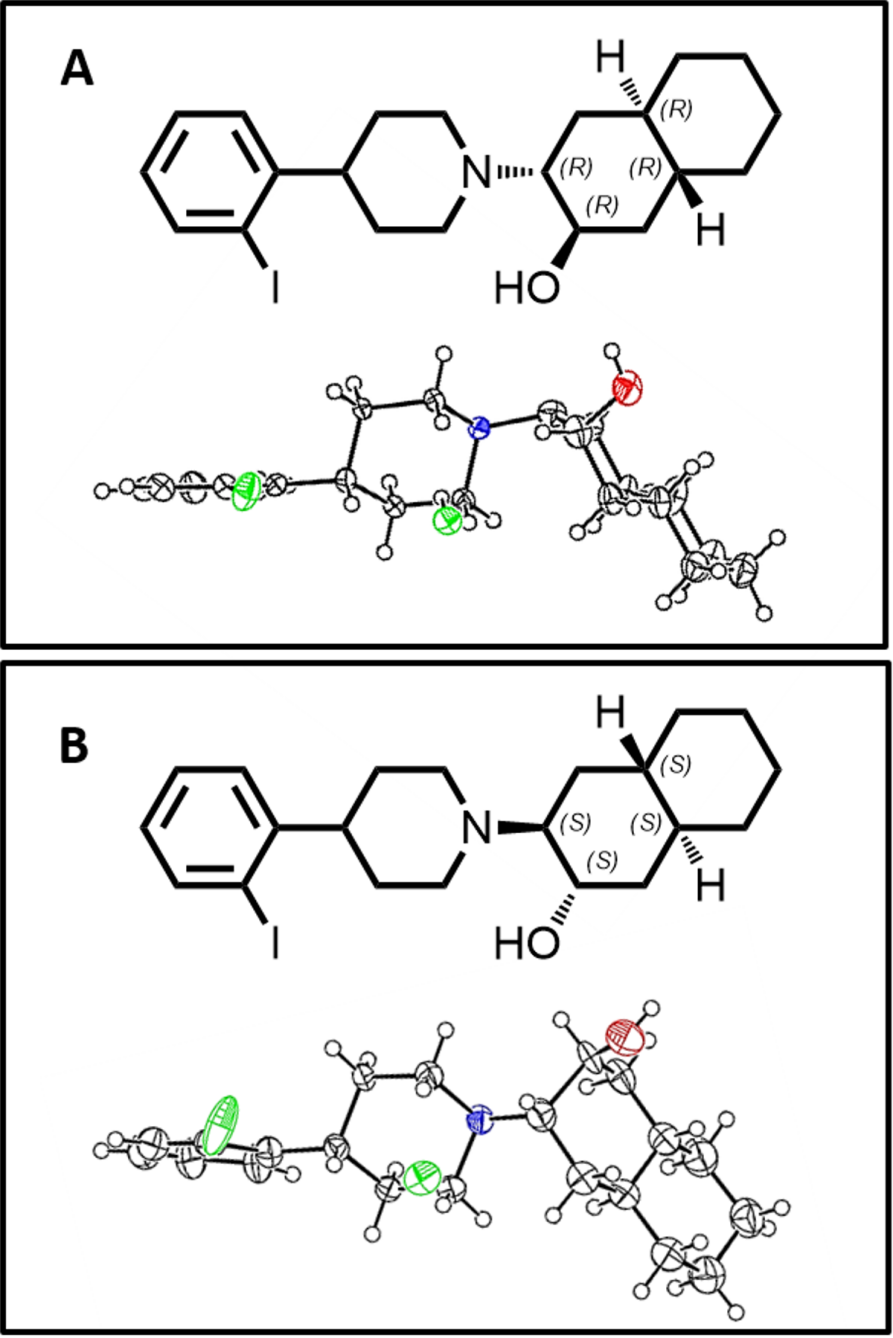
Chemical structure and X-ray crystal structure of (-)-OIDV and (+)-OIDV. A:2R,3R,4aR,8aR)-3-(4-(2-iodophenyl)piperidin-1-yl)decahydronaphthalen-2-ol ((-)-OIDV). B: (2S,3S,4aS,8aS)-3-(4-(2-iodophenyl)piperidin -1-yl)decahydronaphthalen-2-ol ((+)-OIDV).

The HPLC column (Chiralpak IA, 9.6 mm × 250 mm) was purchased from DAICEL Co. (Osaka, Japan). The reverse phase HPLC column (Zorbax-ODS RX-18, 9.6 mm × 250 mm) was purchased from Agilent Technologies Inc. (Santa Clara, CA). [^125^I]NaI was purchased from PerkinElmer Inc. (Waltham, MA)). [^123^I]NaI was supplied by Nihon Medi-Physics Co (Nishinomiya Japan). Specific rotation was obtained on a Nippon Bunko DIP-181 digital polarimeter. Sprague-Dawley (SD) rats were purchased from Sankyo Labo Service Co. (Tokyo, Japan). Animal experiments were performed in compliance with the Guidelines for the Care and Use of Laboratory Animals at the Takara-machi Campus of Kanazawa University. The animal experimental protocols used were approved by the Committee on Animal Experimentation of Kanazawa University (Permit Number: AP-153454). In animal studies, the animals were sacrificed by decapitation under ether anesthesia. The structures of the two OIDV optical isomers were determined by X-ray crystallographic analysis (APEX II ULTRA, Bruker AXS K. K. Japan) performed by Bruker AXS K. K. company (Fig 1) (S1 and S2 Files).

### Isolation of the OIDV optical isomers

Racemic OIDV was separated into its two optical isomers using a normal phase HPLC column (Chiralpak IA, 9.6 mm × 250 mm), with a mobile phase of hexane/dichloromethane/triethylamine (80/20/0.1) at a flow rate of 1.9 mL/min at 35°C. The UV detector wavelength was set to 230 nm. Two major peaks of equal magnitude were separated, with the first peak (retention time: 23.7 min) as (-)-OIDV ([α]$\overset{19}{\text{D}}$ = -13.2 (c = 0.67, chloroform)), and the second peak (retention time: 27.0 min) as (+)-OIDV ([α]$\overset{19}{\text{D}}$ = + 14.5 (c = 0.67, chloroform)), determined by polarimetric analysis.

### Radiosynthesis and Isolation of [^125^I]OIDV optical isomers

[^125^I]OIDV was prepared from o-trimethylstannyl-trans-decalinvesamicol (OTDV) and [^125^I]NaI by the iodo-destannylation reaction under no-carrier-added conditions[32]. (-)-[^125^I]OIDV and (+)-[^125^I]OIDV were separated using a normal phase HPLC column (Chiralpak IA, 9.6 mm × 250 mm) with a mobile phase of hexane/dichloromethane/triethylamine (80/20/0.1) at a flow rate of 1.9 mL/min at 40°C, following purification with a reverse phase HPLC column (Zorbax-ODS RX-C18, 9.6 mm × 250 mm), with a mobile phase of acetonitrile/H_2_O/monoethanolamine (90/10/0.2) at a flow rate of 4.0 mL/min at 40°C. The retention times of (-)-[^125^I]OIDV and (+)-[^125^I]OIDV were 25 min and 28 min, respectively. The radiochemical yield of (-)-[^125^I]OIDV and (+)-[^125^I]OIDV was 37%, and 39%, respectively. The radiochemical purity of both two [^125^I]OIDV isomers was > 99%.

### Radiosynthesis of (-)-[^123^I]OIDV

Racemic o-trimethylstannyl-trans-decalinvesamicol (OTDV) was separated into its two optical isomers using a normal phase HPLC column (Chiralpak IA, 9.6 mm × 250 mm), with a mobile phase of hexane/dichloromethane/triethylamine (90/10/0.1) at a flow rate of 1.9 mL/min at 35°C. To a solution of HCl (0.5 N, 10 μL) and (-)-OTDV (2 mg/mL, 25 μL) in a vial, [^123^I]NH_4_I (111 MBq/150 μL) and 30% H_2_O_2_ (10 μL) were added. The reaction mixture was shaken at room temperature for 20 min. Quenching, neutralization and purification was performed by the same method reported previously[32]. The radiochemical yield of (-)-[^123^I]OIDV was 88%. The radiochemical purity of (-)-[^123^I]OIDV was > 96%.

### Tissue preparations

Rat brain and liver tissue preparations were prepared from dissected brains (not including the cerebellum) and livers from male Sprague-Dawley rats (250–300 g), as previously described[34].

### In vitro competitive binding study

#### VAChT binding

Binding assay was performed as reported previously[34]. Briefly, (-)-[^3^H]vesamicol (K_d_ = 7.40 nM) was used as a radioligand. Various concentrations of (-)-OIDV, (+)-OIDV), decalinvesamicol or vesamicol (from 10^−10^ to 10^−5^ M) were added to rat brain preparations (430–480 μg protein) on ice, and then incubated at 37°C for 60 min in the presence of 200 nM 1,3-di-*o*-tolylguanidine (DTG) to mask the sigma receptors (σ-1 and σ-2). The incubated samples were collected by rapid filtration through Whatman GF/F glass fiber filters presoaked in 0.3% polyethylenimine using a cell harvester. The filters were washed three times with 5 mL of 50 mM Tris-HCl buffer (pH 7.8). Nonspecific binding was determined in the presence of 10 μM (-)-vesamicol. Radioactivity retained on the filters was measured using a liquid scintillation counter (Aloka, LSC-5100).

#### σ-1 receptor binding

Rat cerebrum preparations (430–480 μg protein) were incubated in quadruplicate with 5 nM (+)-[^3^H]pentazocine (K_d_ = 19.9 nM) and various concentrations of (-)-OIDV, (+)-OIDV), decalinvesamicol or vesamicol (from 10^−10^ to 10^−5^ M), or with sigma receptor ligands, in 0.5 ml of 50 mM Tris-HCl (pH 7.8) for 90 min at 37°C. Nonspecific binding was determined in the presence of 10 μM (+)-pentazocine. The incubated samples were treated in the same manner as described for the VAChT binding assays, except that Whatman GF/B glass fiber filters were used.

#### σ-2 receptor binding

Rat liver preparations (about 100 μg protein) were incubated in quadruplicate with 5 nM [^3^H]DTG (K_d_ = 22.3 nM) and various concentrations of (-)-OIDV, (+)-OIDV, decalinvesamicol or vesamicol (from 10^−10^ to 10^−5^ M), or with sigma receptor ligands, in 0.5 ml of 50 mM Tris- HCl (pH 7.8) for 90 min at 37°C in the presence of 1 μM (+)-pentazocine to mask the σ-1 sites. Nonspecific binding was determined in the presence of 10 μM DTG and 1 μM (+)-pentazocine. The incubated samples were treated in the same manner as described for the σ-1 receptor binding assay.

### Data analysis

K_i_ values were calculated using Graphpad Prism (GraphPad Software, Inc. San Diego, USA).

### Biodistribution study

Three groups of male Sprague-Dawley (SD) rats (n = 4 in each group), weighing 250–300 g, were anesthetized with ether and given an intravenous (i.v.) injection of (-)-[^125^I]OIDV or (+)-[^125^I]OIDV (0.4 mL, 185 kBq). At 2, 30, and 60 min post-injection, the animals were sacrificed by decapitation under ether anesthesia. The organs of interest were dissected, weighed, and the radioactivity levels were measured in a gamma scintillation counter (AccuFLEX γ7010, Aloka, Tokyo). The degree of accumulation of radiotracer was expressed as a percentage of the injected dose per gram of tissue (% ID/g).

### *In vivo* blocking study

To evaluate the *in vivo* uptake of (-)-[^125^I]OIDV and (+)-[^125^I]OIDV in the brain, four groups of male SD rats (n = 4 in each group), weighing 250–300 g, received an intravenous injection of either (-)-[^125^I]OIDV or (+)-[^125^I]OIDV (0.4 mL, 185 kBq) alone (control) or with 0.25μmol (+/–)-vesamicol, 0.25 μmol (+)-pentazocine, or 0.25 μmol (+)-3-(3-hydroxyphenyl)-N-propylpiperidine ((+)-3-PPP). (+)-3-PPP was used as σ-1 and σ-2 receptor ligand instead of DTG, because (+)-3-PPP penetrates the blood–brain barrier (BBB) *in vivo* [36–38] (S1 Table).

The rats were sacrificed 60 minutes after injection and their brains collected. The cortex, striatum, cerebellum, and the remainder of the brain were dissected and separated, and their weights and radioactivity measured.

### *Ex vivo* autoradiography

Four SD rats were injected intravenously with either (-)-[^125^I]OIDV or (+)-[^125^I]OIDV (0.4 mL, 1.85 MBq) either alone as a control or with 0.25 μmol (+/–)-vesamicol via the tail vein. At 60 min post-injection, the rats were sacrificed by exsanguination, and perfused via the left ventricle with saline solution (50 mL) followed by 4% paraformaldehyde (pH 7.4, 0.1 M phosphate buffer, 100 mL). Whole brains were removed, frozen in embedding medium at -78°C and cut into 20 μm sections at -25°C using a cryostat microtome. The sections were apposed to an imaging plate (Fujifilm, BAS-IP SR 2025) for eight days. The imaging plates were scanned by a BAS-5000 phosphor image reader (Fujifilm). *Ex vivo* autoradiograhy of (-)-[^123^I]OIDV(0.4 mL, 111 MBq) was performed by the same method of two [^125^I]OIDV isomers.

### Statistical analysis

The results of biodistribution study were statistically analyzed using one-way ANOVA (non-parametric) followed by a Mann Whitney test. Statistical comparisons for the in vivo blocking experiments were performed using one-way ANOVA (non-parametric), Kruskal-Wallis test, and Dunn’s Multiple Comparison test.

## Results

Fig 1 shows the absolute configuration of the enantiomers of OIDV determined by X-ray crystallographic analysis.

### In vitro competitive binding study

Binding affinity (K_i_) of (-)-OIDV, (+)-OIDV, decalinvesamicol, and reference compounds to the VAChT binding sites and sigma receptors (σ-1, σ-2) are shown in Table 1. (-)-OIDV showed a higher affinity for VAChT than (+)-OIDV. (+)-OIDV showed lower affinity for the sigma receptors (σ-1, σ-2) than (-)-OIDV. (-)-OIDV bound to VAChT more selectively than (+/-)-vesamicol.

**Table 1:** Binding Affinities of the OIDV optical isomers to VAChT, and the σ-1 and σ-2 receptors.

Compounds		*K*_i_ (nM)	
	VAChT	σ-1	σ-2
(-)-OIDV	22.1 ± 4.3	168 ± 47.2	59.9 ± 10.6
(+)-OIDV	79.0 ± 19.9	316 ± 26.3	101 ± 18.8
DV	8.6 ± 2.5	74.4 ± 12.0	65.9 ± 12.1
(+/-)-vesamicol	23.3 ± 3.3	19.4 ± 3.0	70.0 ± 16.6
(+)-pentazocine	-	8.7 ± 1.9	2210 ± 653
DTG	-	94.3 ± 19.4	36.5 ± 4.4

*K*_i_ = IC_50_ (1+C/*K*_d_), C = Concentration of radioligand.

Values are the mean ± standard deviation.

### *In vivo* biodistribution

Table 2 shows the tissue distribution of (-)-[^125^I]OIDV and (+)-[^125^I]OIDV at 2 min, 30 min, and 60 min postinjection in the SD rats. No significant differences in intracerebral distribution were observed 2 minutes post-injection. The accumulation of (-)-[^125^I]OIDV and (+)-[^125^I]OIDV in the cerebral cortex was 0.55 ± 0.11%ID/g and 0.56 ± 0.16%ID/g, respectively. (-)-[^125^I]OIDV accumulation in the brain was highest at 30 minutes post-injection. On the other hand, accumulation of (+)-[^125^I]OIDV decreased over time. At 60 min postinjection, (+)-[^125^I]OIDV accumulation in the cerebral cortex decreased to 0.31%ID/g. The accumulatiom of radioactivity in cerebral cortex and striatum, at 30min and 60min post-injection was statistically different between (-)-[^125^I]OIDV and (+)-[^125^I]OIDV. (+)-[^125^I]OIDV showed a higher uptake in the blood, heart, lungs, spleen, kidneys, and liver than (-)-[^125^I]OIDV at 2 min postinjection. In particular, (+)-[^125^I]OIDV showed more accumulation in the lung (9.82 ± 1.78%ID/g) at 2 min post-injection compared with (-)-[^125^I]OIDV (5.84 ± 0.44%ID/g).

**Table 2:** Biodistribution of (-)-[^125^I]OIDV and (+)-[^125^I]OIDV in rats.

Organs	(-)-^125^I-OIDV			(+)-^125^I-OIDV		
	% ID/g			% ID/g		
	Time post-injection			Time post-injection		
	2 min	30 min	60 min	2 min	30 min	60 min
**Blood**	**0.13 ± 0.01**	**0.05 ± 0.01**	**0.04 ± 0.01**	**0.22 ± 0.03**	**0.06 ± 0.00**	**0.05 ± 0.00**
**Heart**	**2.34 ± 0.23**	**0.44 ± 0.06**	**0.25 ± 0.05**	**2.79 ± 0.97**	**0.45 ± 0.04**	**0.27 ± 0.01**
**Lung**	**5.84 ± 0.44**	**2.51 ± 0.90**	**1.54 ± 0.41**	**9.82 ± 1.78**	**2.59 ± 0.38**	**1.32 ± 0.08**
**Pancreas**	**2.66 ± 0.40**	**4.67 ± 0.58**	**4.21 ± 0.20**	**2.54 ± 0.57**	**3.51 ± 0.30**	**2.81 ± 0.49**
**Spleen**	**0.94 ± 0.40**	**1.37 ± 0.27**	**0.91 ± 0.08**	**1.51 ± 0.37**	**2.33 ± 0.25**	**1.99 ± 0.18**
**Kidney**	**2.84 ± 0.63**	**2.35 ± 0.38**	**2.21 ± 0.17**	**3.46 ± 0.76**	**2.25 ± 0.07**	**1.47 ± 0.06**
**Small intestine**	**0.89 ± 0.12**	**1.45 ± 0.50**	**1.85 ± 0.14**	**1.15 ± 1.31**	**1.68 ± 0.13**	**1.60 ± 0.29**
**Stomach**	**0.18 ± 0.09**	**0.22 ± 0.23**	**0.24 ± 0.22**	**0.17 ± 0.07**	**0.78 ± 0.73**	**0.29 ± 0.33**
**Liver**	**2.07 ± 0.63**	**2.60 ± 0.75**	**2.19 ± 0.17**	**2.20 ± 0.22**	**2.74 ± 0.20**	**1.94 ± 0.22**
**Cortex**	**0.55 ± 0.10**	0.62 ± 0.10*	**0.51 ± 0.06**^**!**^	**0.56 ± 0.16**	0.46 ± 0.07*	**0.31 ± 0.02**^**!**^
**Striatum**	**0.47 ± 0.10**	**0.63 ± 0.09**^**#**^	**0.50 ± 0.07**^**$**^	**0.49 ± 0.15**	**0.45 ± 0.07**^**#**^	**0.31 ± 0.02**^**$**^
**Cerebellum**	**0.53 ± 0.04**	**0.55 ± 0.11**	**0.41 ± 0.07**	**0.54 ± 0.15**	**0.46 ± 0.08**	**0.36 ± 0.05**
**Rest brain**	**0.48 ± 0.07**	**0.55 ± 0.09**	**0.47 ± 0.06**^**+**^	**0.50 ± 0.13**	**0.44 ± 0.06**	**0.32 ± 0.02**^**+**^

Values are mean ± standard deviation (SD) of four rats (n = 4) at each time post-injection

*,!,#,$,+: A one-way ANOVA followed by a Mann Whitney test was performed by GraphPad Prism Version 4 software, compared with the control. P <0.05 (n = 4).

### *In vivo* blocking study

**Fig 2:**
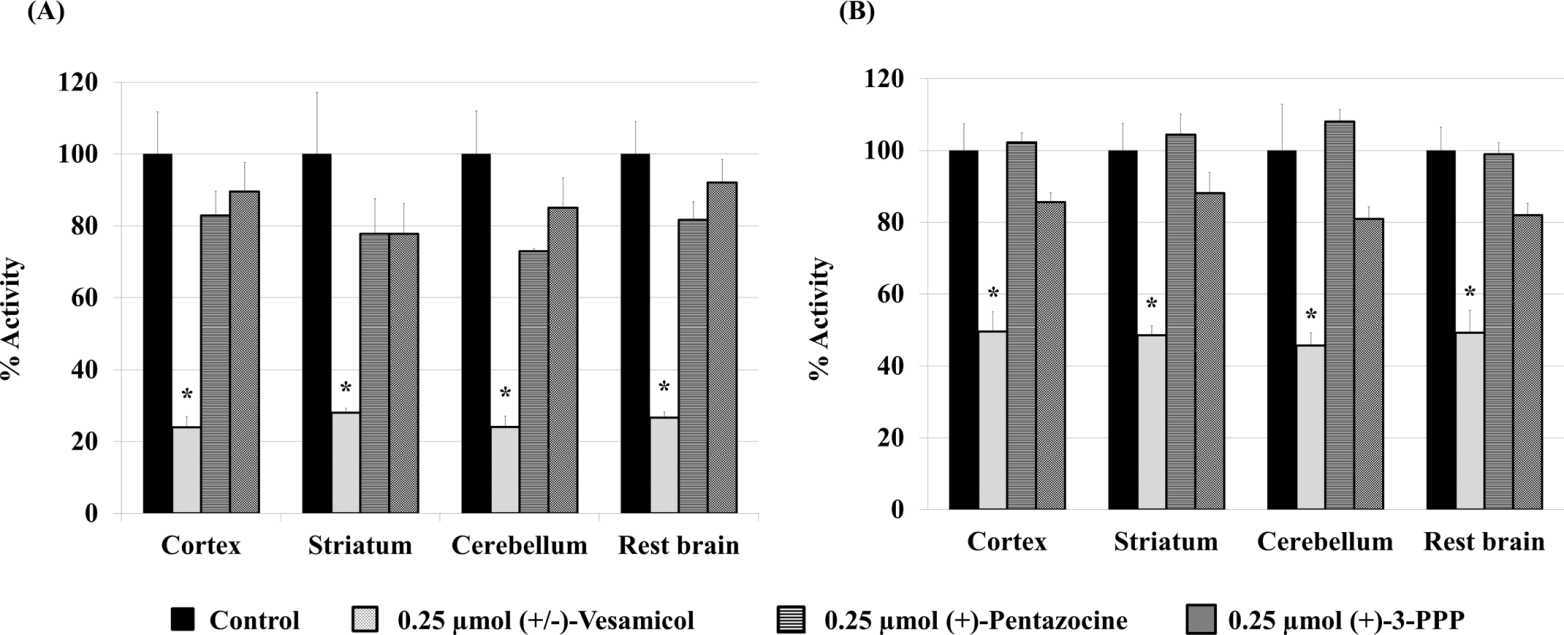
Uptake inhibition of (-)-[^125^I]OIDV (A) and (+)-[^125^I]OIDV (B). The vertical axis shows the mean radioactivity signal in the brain region (cerebral cortex, striatum, cerebellum, and the remainder) of each group injected with either (-)-[^125^I]OIDV or (+)-[^125^I]OIDV alone (control) or with (+/-)-vesamicol (0.250 µmol), (+)-pentazocine (0.250 µmol), or (+)-3-PPP (0.250 µmol). Uptake of (-)-[^125^I]OIDV or (+)-[^125^I]OIDV alone was arbitrarily set to 100%. A one-way ANOVA followed by a Kruskal-Wallis test, and Dunn’s Multiple Comparison test was performed by GraphPad Prism Version 4 software, compared with the control. Here, *P <0.01.

To investigate the binding characteristics of (-)-[^125^I]OIDV and (+)-[^125^I]OIDV *in vivo*, we studied the blocking effect of three agents [(+/–)-vesamicol (VAChT ligand), (+)-pentazocine (σ-1 receptor ligand), or (+)-3-PPP (σ-1, σ-2 receptor ligands)] on the regional brain uptake of (-)-[^125^I]OIDV and (+)-[^125^I]OIDV (Fig 2). The uptake of (-)-[^125^I]OIDV was remarkably decreased (approximately 25% of control) in all four brain regions investigated with co-injection of vesamicol, compared to (+)-[^125^I]OIDV (approximately 50% of control). On the other hand, co-injection of (+)-pentazocine or (+)-3PPP only slightly reduced the uptake of (-)-[^125^I]OIDV in all four brain regions (72–82% of control). No decrease in uptake of (+)-[^125^I]OIDV with co-injection of (+)-pentazocine was observed. Co-injection of (+)-3PPP slightly reduced the uptake of (+)-[^125^I]OIDV in all four brain regions (81–88% of control).

### *Ex vivo* autoradiography

**Fig 3:**
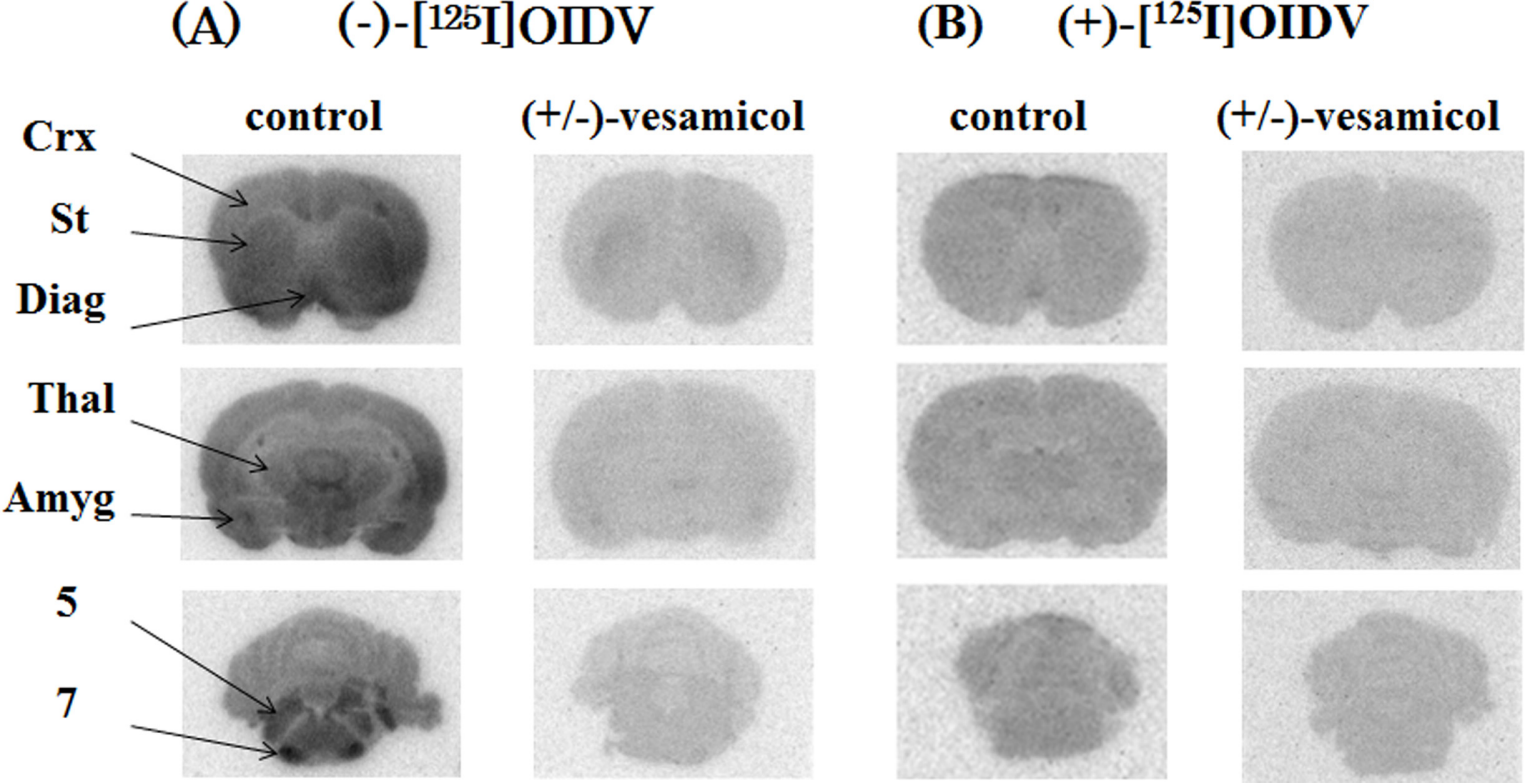
*Ex vivo* autoradiograms of (-)-[^125^I]OIDV and (+)-[^125^I]OIDV in the rat brain 60 min post-injection with (-)-[^125^I]OIDV or (+)-[^125^I]OIDV alone or with 0.250 μmol (+/-)-vesamicol. Abbreviations: Crx: Cortex, St: Striatum, Diag: Diagonal band, Thal: Thalamus, Amyg: Amygdaloid nucleus, 5: Trigeminal nucleus,7: Facial nucleus.

**Fig 4:**
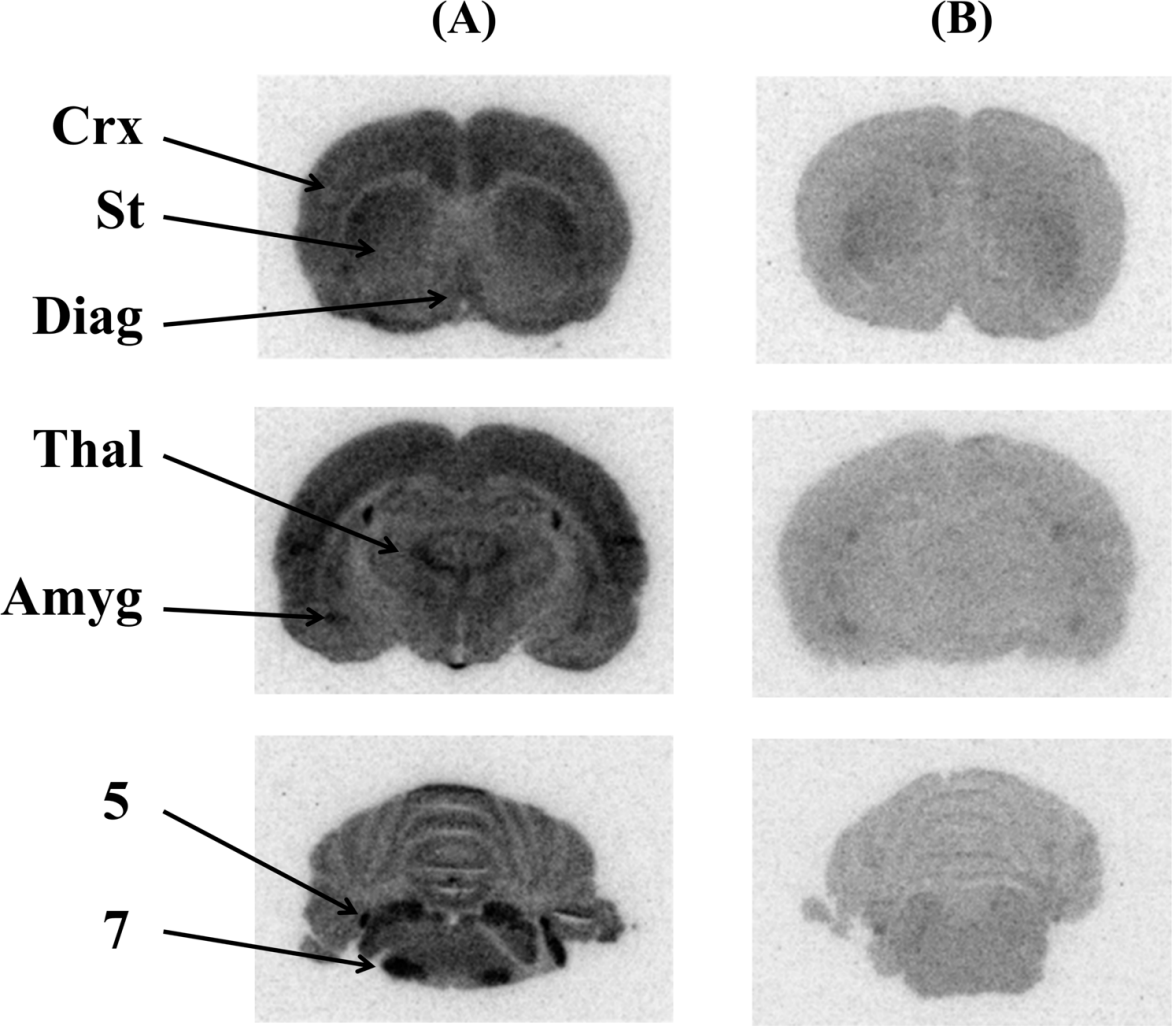
Ex vivo autoradiograms of the rat brain 60 min post-injection of (-)-[^123^I]OIDV alone (A); or with 0.250 μmol (±)-vesamicol as an inhibitor (B). Abbreviations: Crx: Cortex, St: Striatum, Diag: Diagonal band, Thal: Thalamus, Amyg: Amygdaloid nucleus, 5: Trigeminal nucleus, 7: Facial nucleus.

Fig 3 shows coronal images of rat brains visualized by *ex vivo* autoradiography with (-)-[^125^I]OIDV or (+)-[^125^I]OIDV 60 min after injection. (-)-[^125^I]OIDV was distributed in characteristically VAChT-rich regions, such as the cortex, striatum, diagonal band, amygdaloid nucleus, and trigeminal and facial nucleus. This accumulation of (-)-[^125^I]OIDV was remarkably decreased with co-injection of 0.25 μmol vesamicol. On the other hand, accumulation of (+)-[^125^I]OIDV in characteristically VAChT-rich regions was not observed, and radioactive signal of (+)-[^125^I]OIDV was uniformly low throughout the entire rat brain. Fig 4 shows coronal images of rat brains visualized by *ex vivo* autoradiography with (-)-[^123^I]OIDV, showing the same regional distribution as that of (-)-[^125^I]OIDV.

## Discussion

OIDV was separated into its two optical isomers, (-)-OIDV and (+)-OIDV, with high purity using HPLC with a normal phase column Chiralpak IA (DAICEL Co., Japan). The structures of the two isomers were determined by X-ray crystallographic analysis, and showed that a hydroxyl group at the 2-position and a hydrogen at the 10-position of decalin, located close to the hydroxyl group, were arranged in *cis* configuration (Fig 1).

(-)-OIDV showed a higher binding affinity for VAChT than (+)-OIDV under *in vitro* condition. The binding selectivity of (-)-OIDV to VAChT was superior to that of (+)-OIDV, although the binding affinity of (+)-OIDV to the sigma receptors was lower than (-)-OIDV (Table 1). Uptake of (-)-[^125^I]OIDV in the cerebral cortex was 0.55 ± 0.10, 0.62 ± 0.10, and 0.51 ± 0.06%ID/g at 2, 30, and 60 min post-injection, respectively. Uptake of (-)-[^125^I]OIDV in all regions (cerebral cortex, striatum, cerebellum, and the remainder) was highest 30 min post-injection. The long retention (greater than 60 min) of the accumulated (-)-[^125^I]OIDV, and the low level of radioactivity detected in the blood may be advantageous for VAChT imaging for SPECT (Table 2). On the other hand, the uptake of (+)-[^125^I]OIDV in the cerebral cortex was 0.56 ± 0.16%ID/g 2 min post-injection and 0.32 ± 0.02%ID/g 60 min post-injection, demonstrating the rapid clearance of (+)-[^125^I]OIDV from rat brain *in vivo* (Table 2). Two isomers of [^125^I]OIDV showed the same regional brain distribution 2 min post-injection, because regional brain distribution of two optical isomers reflected more strongly blood flow than VAChT density. However, at 30 min and 60 min post-injection, (-)-[^125^I]OIDV showed 1.4–1.6 times higher striatum distribution than (+)-[^125^I]OIDV. Other regional brain distribution of (-)-[^125^I]OIDV differed also clearly with those of (+)-[^125^I]OIDV. Thus, (-)-[^125^I]OIDV bound to VAChT with higher affinity than (+)-[^125^I]OIDV. However, the difference of binding property of [^125^I]OIDV was fewer than that of [^11^C]HATP[35] *in vivo*. That will be because the difference of binding affinity of two optical isomers of [^125^I]OIDV to VAChT was fewer than that of [^11^C]HATP. In our previous paper, 0.250 μmol of vesamicol blocked more strongly than 0.125 μmol of vesamicol. 0.125 μmol of (+)-pentazocine and (+)-3-PPP was considered to be not enough concentration to blocked sigma-1 and/or sigma-2 receptor in *in vivo* blocking study. In this study, 0.250 μmol of inhibitors were used to *in vivo* blocking study[33]. In the *in vivo* blocking study, vesamicol inhibited brain uptake of (-)-[^125^I]OIDV (decrease in uptake of 71–73%) to a greater extent than (+)-[^125^I]OIDV uptake (decrease in uptake of 50–55%). The inhibition of (-)-[^125^I]OIDV brain uptake by the sigma ligands (pentazocine, 3-PPP) was also more pronounced than inhibition of (+)-[^125^I]OIDV uptake, however this difference was not statistically significant. The decrease of regional brain accumulation of two [^125^I]OIDV isomers by 0.250 μmol of (+)-pentazocine and (+)-3PPP was greater than that of racemic [^125^I]OIDV by 0.125 μmol of (+)-pentazocine and (+)-3PPP[33], which might show that regional brain accumulation of [^125^I]OIDV decreases depending on the concentration of sigma ligands. However, the decrease of regional brain accumulation of two [^125^I]OIDV isomers by 0.250 μmol of (+)-pentazocine and (+)-3PPP was not statistically significant. Thus, the *in vivo* accumulation of (-)-[^125^I]OIDV in the brain appears to be due to the selective binding of (-)-[^125^I]OIDV to VAChT, although binding to the sigma receptors cannot be entirely dismissed. The decrease in accumulation of (+)-[^125^I]OIDV in the brain by co-administration of vesamicol was weak due in part to the high non-specific binding and quick clearance of (+)-[^125^I]OIDV from the brain, as well as a relatively low affinity of (+)-[^125^I]OIDV to VAChT (Fig 2). In vitro and *in vivo* characteristics of (-)-[^125^I]OIDV was not significantly different from that of racemic [^125^I]OIDV[33] in experiments using normal animal. However, in experiments using genetically modified mice or in clinical application in future, some difference between (-)-[^125^I]OIDV and racemic [^125^I]OIDV will be observed, because unlike racemic [^125^I]OIDV, (-)-[^125^I]OIDV did not include (+)-[^125^I]OIDV disturbing the *in vivo* VAChT accumulation of [^125^I]OIDV in the brain.

The present study did not perform *in vivo* metabolite analysis, because we were unable to observe the presence of radiolabeled metabolites in the brain derived from racemic [^125^I]OIDV in our previous report^33)^. The metabolic process of both of the OIDV optical isomers appeared to be similar, with similar increases in radioactivity in the pancreas, small intestines, and liver.

In the *ex vivo* autoradiographic experiments (Figs 3 and 4), brain distribution of (-)-[^125^I]OIDV, particularly in the cerebral cortex, lateral striatum, diagonal band, thalamus, amygdaloidal nucleus, cerebellum, and nuclei of the cranial nerves, was similar to the brain distribution of (-)-[^3^H]vesamicol in rats *ex vivo*[39]. We also synthesized (-)-[^123^I]OIDVsuitable for clinical application. (-)-[^123^I]OIDV for SPECT showed the same regional brain distribution as (-)-[^125^I]OIDV in the *ex vivo* autoradiographic study.

Regional brain distribution of (-)-[^3^H]vesamicol was shown to be similar to that of [^3^H]hemicholinium-3, which itself has been shown to have a high binding affinity for choline transporter (ChT) by *in vitro* autoradiography, localized to the pre-synapse of cholinergic nerve terminals[40,41]. Signaling in the cholinergic system utilizing ACh as neurotransmitter involves the muscarinic acetylcholine receptor (mAChR) (M_1-5_) and nicotinic acetylcholine receptor systems. A high density of muscarinic acetylcholine M_1_ receptor is found in forebrain areas including the cerebral cortex, striatum, hippocampus, and amygdala, and the muscarinic acetylcholine M_2_ receptor in the anterior & intralaminar nuclei of the thalamus, all motor nuclei of the cranial nerves, and the granule and Purkinje cell layers of the cerebellum[42,43]. The M_1_, M_2_, and M_4_ muscarinic acetylcholine receptors are differentially localized in the striatum[44], and the diagonal band has been shown to be a muscarinic acetylcholine M_2_ –M_5_ receptor-abundant area[45]. The nicotinic receptor is widely distributed in the anteroventral nucleus of the thalamus[46]. Therefore, it is thought that VAChT, localized in the pre-synapses of both the muscarinic nerve systems (M1 –M5 receptors) and the nicotinic nerve systems, is widely distributed throughout various regions of the brain, including the cerebral cortex, striatum, diagonal band, hippocampus, thalamus, amygdaloidal nucleus, cerebellum, and nuclei of the cranial nerves. Due to the similar regional distribution of (-)-[^125^I/^123^I]OIDV, this may reflect the VAChT-rich regions of the rat brain. However, various VAChT radioligands including IBVM [47], MIBT[23][^18^F]FEOBV [25], [^18^F]FBMV [48] or [^18^F]FBT [24] accumulated in striatum much higher levels than in cerebral cortex. The uptake ratio of striatum to cerebral cortex of those VAChT radioligands was different from that of (-)-[^125^I/^123^I]OIDV. Expressions of VAChT in brain were characterized by higher concentration of VAChT in striatum than cerebral cortex, by regional brain distribution of VAChT imaging ligands and [^3^H]vesamicol in *in vivo* or *in vitro* [26,41,47]. However, cholinergic neurons in striatum are not necessarily related to cognitive impairment in Alzheimer’s disease because cholinergic neurons in striatum consist of local circuit cells, which mean a nerve signal is transmitted only in striatum. On the other hand, because cholinergic neurons in cerebral cortex belong to projection neurons which the basal forebrain cholinergic neuron complex such as the nucleus basalis of Meynert (NMB) [49,50], the medial septal nucleus and the diagonal band nuclei projects to, and the function of cerebral cortex is associated with a cognitive, learning and memory functions, VAChT in cerebral cortex will be suitable to the target for early diagnosis of Alzheimer’s disease. On the other hand, because cholinergic neurons in striatum consist of local circuit cells, which mean a nerve signal is transmitted only in striatum, cholinergic neurons in striatum are not necessarily related to cognitive impairment in Alzheimer’s disease.

## Conclusion

(-)-OIDV, one of the optical isomers of OIDV, showed higher binding affinity and selectivity to VAChT in comparison with (+)-OIDV in vitro. In vivo, (-)-[^125^I]OIDV was distributed in regions of the rat brain thought to be VAChT-rich. (-)-[^123^I]OIDV suitable for clinical application was able to be easily synthesized and purified using (-)-OTDV as precursor. (-)-[^123^I]OIDV may be a suitable radioligand for the study of dementia, which is characterized by the degeneration of the cholinergic neurotransmitter system.

## Supporting Information

S1 File Crystal data and structure refinement for (-)-OIDV. (PDF)

S2 File Crystal data and structure refinement for (+)-OIDV. (PDF)

S1 Table The regional biodistribution of (+)-[^3^H]-3-PPP in rat’s brain. (DOC)
