# Supplementary material for: In Vivo Differences between Two Optical Isomers of Radioiodinated o-iodo-trans-decalinvesamicol for Use as a Radioligand for the Vesicular Acetylcholine Transporter
Source: PLoS One. 2016 Jan 11;11(1):e0146719. doi: 10.1371/journal.pone.0146719 (PMC4713475; doi:10.1371/journal.pone.0146719)
Supplement: S1 File — (PDF) [file pone.0146719.s001.pdf]

Crystal Structure report for (-)-OIDV

Computing details

Data collection: *APEX2* v2013 (Bruker AXS Inc.); cell refinement: *APEX2* v2013 (Bruker AXS); data reduction: *SAINT* v8.3C (Bruker AXS Inc.); program(s) used to refine structure: *SHELXL2013* (Sheldrick, 2013); molecular graphics: XSHELL v6.3.1 (Bruker AXS Inc.); software used to prepare material for publication: Generate Report (Bruker AXS Inc.).

Crystal data

$C_{22}H_{28}INO$	$Z = 4$
$M_r = 449.35$	$F(000) = 912$
Orthorhombic, $P2_12_12_1$	$D_x = 1.540$ Mg m^{-3}
$a = 9.982$ (2) Å	Mo $K\alpha$ radiation, $\lambda = 0.71073$ Å
$b = 13.149$ (3) Å	$\mu = 1.66$ mm^{-1}
$c = 14.770$ (3) Å	$T = 93$ K
$V = 1938.6$ (8) Å^3	0.09 × 0.07 × 0.05 mm

Data collection

Bruker D8 goniometer diffractometer	3524 independent reflections
Radiation source: rotating-anode X-ray tube	3143 reflections with $I > 2\sigma(I)$
Multilayered conforcal mirror monochromator	$R_{int} = 0.060$
phi and ω scans	$\theta_{max} = 25.3°$, $\theta_{min} = 2.1°$
Absorption correction: multi-scan *SADABS* Sheldrick 1996	$h = -11 \rightarrow 12$
$T_{min} = 0.86$, $T_{max} = 0.92$	$k = -15 \rightarrow 11$
9534 measured reflections	$l = -16 \rightarrow 17$

Refinement

Refinement on F^2	Hydrogen site location: mixed
Least-squares matrix: full	H atoms treated by a mixture of independent and constrained refinement
$R[F^2 > 2\sigma(F^2)] = 0.038$	$w = 1/[\sigma^2(F_o^2)]$ where $P = (F_o^2 + 2F_c^2)/3$
$wR(F^2) = 0.061$	$(\Delta/\sigma)_{max} < 0.001$
$S = 1.00$	$\Delta\rho_{max} = 0.58$ e Å^{-3}
3524 reflections	$\Delta\rho_{min} = -0.41$ e Å^{-3}
221 parameters	Absolute structure: Flack x determined using 1227 quotients [(I+)-(I-)]/[(I+)+(I-)] (Parsons and Flack (2004), Acta Cryst. A60, s61).
162 restraints	Absolute structure parameter: 0.03 (3)

Special details

Geometry. All esds (except the esd in the dihedral angle between two l.s. planes) are estimated using the full covariance matrix. The cell esds are taken into account individually in the estimation of esds in distances, angles and torsion angles; correlations between esds in cell parameters are only used when they are defined by crystal symmetry. An approximate (isotropic) treatment of cell esds is used for estimating esds involving l.s. planes.

Fractional atomic coordinates and isotropic or equivalent isotropic displacement parameters (Å^2)

	x	*y*	*z*	U_{iso}*/U_{eq}
C1	-0.1613 (6)	0.1229 (5)	0.5603 (4)	0.0092 (14)
C2	-0.2569 (7)	0.1426 (6)	0.4940 (4)	0.0178 (17)
H2A	-0.2704	0.0963	0.4455	0.021*
C3	-0.3324 (7)	0.2314 (6)	0.4998 (5)	0.0191 (18)
H3A	-0.3984	0.2459	0.4553	0.023*
C4	-0.3112 (7)	0.2979 (6)	0.5698 (5)	0.0195 (18)
H4A	-0.3623	0.3587	0.5738	0.023*
C5	-0.2150 (6)	0.2763 (6)	0.6349 (5)	0.0157 (16)
H5A	-0.202	0.323	0.6832	0.019*
C6	-0.1374 (6)	0.1891 (5)	0.6319 (5)	0.0122 (15)
C7	-0.0325 (7)	0.1675 (5)	0.7035 (4)	0.0116 (15)

H7A	0.0438	0.1324	0.6727	0.014*
C8	-0.0850 (6)	0.0954 (5)	0.7766 (4)	0.0127 (16)
H8A	-0.1186	0.0323	0.7479	0.015*
H8B	-0.1608	0.1279	0.8087	0.015*
C9	0.0250 (7)	0.0684 (5)	0.8450 (4)	0.0149 (17)
H9A	-0.0125	0.0225	0.8916	0.018*
H9B	0.0978	0.0317	0.8134	0.018*
C10	0.1318 (7)	0.2286 (6)	0.8182 (5)	0.0157 (17)
H10A	0.2048	0.1939	0.785	0.019*
H10B	0.1703	0.2899	0.8472	0.019*
C11	0.0248 (6)	0.2615 (5)	0.7509 (5)	0.0112 (16)
H11A	0.0642	0.3081	0.7056	0.013*
H11B	-0.0477	0.2981	0.783	0.013*
C12	0.1937 (6)	0.1314 (5)	0.9507 (5)	0.0111 (14)
H12A	0.2732	0.1164	0.9118	0.013*
C13	0.1672 (6)	0.0375 (5)	1.0085 (4)	0.0139 (16)
H13A	0.1386	-0.0188	0.9684	0.017*
H13B	0.252	0.0167	1.038	0.017*
C14	0.0611 (8)	0.0531 (5)	1.0813 (4)	0.0152 (15)
H14A	-0.0255	0.0705	1.051	0.018*
C15	0.0412 (7)	-0.0443 (5)	1.1357 (4)	0.0228 (17)
H15A	0.1267	-0.064	1.1647	0.027*
H15B	0.0135	-0.1001	1.0947	0.027*
C16	-0.0672 (7)	-0.0283 (6)	1.2092 (4)	0.0304 (19)
H16A	-0.1546	-0.0143	1.1802	0.036*
H16B	-0.0763	-0.0908	1.2462	0.036*
C17	-0.0278 (8)	0.0611 (6)	1.2700 (5)	0.030 (2)
H17A	-0.1005	0.0739	1.3142	0.035*
H17B	0.054	0.0432	1.3043	0.035*
C18	-0.0024 (7)	0.1563 (7)	1.2165 (5)	0.0234 (19)
H18A	0.0289	0.2105	1.258	0.028*
H18B	-0.0875	0.1794	1.1889	0.028*
C19	0.1006 (6)	0.1412 (6)	1.1427 (5)	0.0145 (16)
H19A	0.1864	0.1217	1.173	0.017*
C20	0.1287 (7)	0.2365 (6)	1.0875 (5)	0.0141 (17)
H20A	0.0438	0.2602	1.0599	0.017*

H20B	0.1609	0.2906	1.1286	0.017*
C21	0.2315 (6)	0.2210 (5)	1.0129 (4)	0.0108 (16)
H21A	0.2401	0.2847	0.9763	0.013*
H1O	0.396 (6)	0.245 (5)	1.065 (4)	0.01 (2)*
I1	-0.05298 (4)	-0.01369 (3)	0.54608 (3)	0.01462 (12)
N1	0.0808 (5)	0.1598 (4)	0.8895 (3)	0.0109 (13)
O1	0.3599 (4)	0.1944 (4)	1.0514 (4)	0.0151 (11)

Atomic displacement parameters (Å^2)

	U^{11}	U^{22}	U^{33}	U^{12}	U^{13}	U^{23}
C1	0.008 (2)	0.011 (2)	0.008 (2)	0.000 (2)	0.003 (2)	0.002 (2)
C2	0.019 (4)	0.024 (4)	0.010 (3)	-0.001 (3)	0.002 (3)	-0.005 (3)
C3	0.015 (4)	0.030 (5)	0.012 (4)	0.004 (4)	-0.003 (3)	0.004 (4)
C4	0.014 (4)	0.020 (4)	0.025 (4)	0.004 (3)	-0.003 (3)	0.005 (3)
C5	0.016 (4)	0.020 (4)	0.011 (3)	0.001 (3)	0.002 (3)	0.002 (3)
C6	0.011 (3)	0.015 (4)	0.011 (3)	-0.003 (3)	0.007 (3)	0.000 (3)
C7	0.010 (3)	0.016 (4)	0.009 (3)	0.004 (3)	-0.001 (3)	-0.005 (3)
C8	0.009 (4)	0.019 (4)	0.011 (3)	-0.002 (3)	-0.001 (3)	-0.004 (3)
C9	0.017 (4)	0.013 (4)	0.014 (3)	-0.002 (3)	0.000 (3)	0.001 (3)
C10	0.018 (4)	0.014 (4)	0.014 (4)	-0.007 (3)	0.000 (3)	-0.002 (3)
C11	0.012 (4)	0.014 (4)	0.008 (3)	-0.002 (3)	-0.001 (3)	0.000 (3)
C12	0.003 (3)	0.016 (3)	0.014 (3)	0.004 (3)	0.003 (3)	0.000 (4)
C13	0.013 (3)	0.013 (4)	0.015 (3)	0.001 (3)	-0.005 (3)	0.002 (3)
C14	0.011 (3)	0.023 (4)	0.012 (3)	-0.006 (4)	0.000 (3)	0.003 (3)
C15	0.025 (4)	0.029 (4)	0.015 (3)	-0.013 (4)	-0.004 (4)	0.006 (3)
C16	0.032 (4)	0.036 (5)	0.023 (4)	-0.012 (4)	0.002 (4)	0.005 (4)
C17	0.024 (4)	0.046 (5)	0.019 (4)	-0.009 (4)	0.005 (3)	0.003 (4)
C18	0.019 (4)	0.035 (5)	0.016 (4)	-0.003 (4)	0.000 (3)	-0.004 (4)
C19	0.007 (3)	0.023 (4)	0.013 (3)	-0.003 (3)	-0.004 (3)	-0.002 (3)
C20	0.012 (4)	0.015 (4)	0.015 (4)	0.004 (3)	-0.007 (3)	-0.002 (3)
C21	0.007 (3)	0.009 (4)	0.016 (4)	0.000 (3)	-0.006 (3)	-0.003 (3)
I1	0.0152 (2)	0.0139 (2)	0.0148 (2)	0.0006 (2)	0.0011 (2)	-0.0025 (2)
N1	0.012 (3)	0.012 (3)	0.009 (3)	0.000 (3)	-0.003 (2)	0.001 (2)
O1	0.010 (2)	0.016 (3)	0.019 (3)	-0.003 (2)	-0.006 (3)	0.000 (3)

Geometric parameters (Å, º) for (I)

C1—C6	1.390 (9)	C12—C21	1.541 (9)
C1—C2	1.391 (9)	C12—H12A	1.0
C1—I1	2.107 (6)	C13—C14	1.523 (9)
C2—C3	1.392 (11)	C13—H13A	0.99
C2—H2A	0.95	C13—H13B	0.99
C3—C4	1.371 (10)	C14—C19	1.524 (9)
C3—H3A	0.95	C14—C15	1.525 (9)
C4—C5	1.388 (9)	C14—H14A	1.0
C4—H4A	0.95	C15—C16	1.547 (9)
C5—C6	1.386 (10)	C15—H15A	0.99
C5—H5A	0.95	C15—H15B	0.99
C6—C7	1.514 (9)	C16—C17	1.531 (10)
C7—C8	1.530 (9)	C16—H16A	0.99
C7—C11	1.532 (10)	C16—H16B	0.99
C7—H7A	1.0	C17—C18	1.502 (11)
C8—C9	1.533 (8)	C17—H17A	0.99
C8—H8A	0.99	C17—H17B	0.99
C8—H8B	0.99	C18—C19	1.512 (9)
C9—N1	1.479 (8)	C18—H18A	0.99
C9—H9A	0.99	C18—H18B	0.99
C9—H9B	0.99	C19—C20	1.520 (10)
C10—N1	1.478 (8)	C19—H19A	1.0
C10—C11	1.522 (10)	C20—C21	1.519 (9)
C10—H10A	0.99	C20—H20A	0.99
C10—H10B	0.99	C20—H20B	0.99
C11—H11A	0.99	C21—O1	1.445 (7)
C11—H11B	0.99	C21—H21A	1.0
C12—N1	1.493 (8)	O1—H1O	0.79 (6)
C12—C13	1.524 (9)		

C6—C1—C2	122.4 (6)	C12—C13—H13A	108.7
C6—C1—I1	121.4 (5)	C14—C13—H13B	108.7
C2—C1—I1	116.2 (5)	C12—C13—H13B	108.7
C1—C2—C3	119.0 (7)	H13A—C13—H13B	107.6
C1—C2—H2A	120.5	C13—C14—C19	110.0 (6)
C3—C2—H2A	120.5	C13—C14—C15	110.5 (6)

C4—C3—C2	119.9 (7)	C19—C14—C15	111.0 (5)
C4—C3—H3A	120.1	C13—C14—H14A	108.4
C2—C3—H3A	120.1	C19—C14—H14A	108.4
C3—C4—C5	119.9 (7)	C15—C14—H14A	108.4
C3—C4—H4A	120.1	C14—C15—C16	110.3 (6)
C5—C4—H4A	120.1	C14—C15—H15A	109.6
C6—C5—C4	122.3 (7)	C16—C15—H15A	109.6
C6—C5—H5A	118.8	C14—C15—H15B	109.6
C4—C5—H5A	118.8	C16—C15—H15B	109.6
C5—C6—C1	116.5 (6)	H15A—C15—H15B	108.1
C5—C6—C7	121.3 (6)	C17—C16—C15	109.7 (6)
C1—C6—C7	122.2 (6)	C17—C16—H16A	109.7
C6—C7—C8	111.8 (6)	C15—C16—H16A	109.7
C6—C7—C11	115.2 (6)	C17—C16—H16B	109.7
C8—C7—C11	107.8 (5)	C15—C16—H16B	109.7
C6—C7—H7A	107.2	H16A—C16—H16B	108.2
C8—C7—H7A	107.2	C18—C17—C16	112.0 (6)
C11—C7—H7A	107.2	C18—C17—H17A	109.2
C7—C8—C9	111.3 (5)	C16—C17—H17A	109.2
C7—C8—H8A	109.4	C18—C17—H17B	109.2
C9—C8—H8A	109.4	C16—C17—H17B	109.2
C7—C8—H8B	109.4	H17A—C17—H17B	107.9
C9—C8—H8B	109.4	C17—C18—C19	112.6 (7)
H8A—C8—H8B	108.0	C17—C18—H18A	109.1
N1—C9—C8	112.0 (6)	C19—C18—H18A	109.1
N1—C9—H9A	109.2	C17—C18—H18B	109.1
C8—C9—H9A	109.2	C19—C18—H18B	109.1
N1—C9—H9B	109.2	H18A—C18—H18B	107.8
C8—C9—H9B	109.2	C18—C19—C20	113.8 (6)
H9A—C9—H9B	107.9	C18—C19—C14	110.7 (6)
N1—C10—C11	113.4 (6)	C20—C19—C14	110.8 (5)
N1—C10—H10A	108.9	C18—C19—H19A	107.1
C11—C10—H10A	108.9	C20—C19—H19A	107.1
N1—C10—H10B	108.9	C14—C19—H19A	107.1
C11—C10—H10B	108.9	C21—C20—C19	113.8 (6)
H10A—C10—H10B	107.7	C21—C20—H20A	108.8

C10—C11—C7	109.4 (6)	C19—C20—H20A	108.8
C10—C11—H11A	109.8	C21—C20—H20B	108.8
C7—C11—H11A	109.8	C19—C20—H20B	108.8
C10—C11—H11B	109.8	H20A—C20—H20B	107.7
C7—C11—H11B	109.8	O1—C21—C20	110.2 (5)
H11A—C11—H11B	108.2	O1—C21—C12	105.4 (5)
N1—C12—C13	114.3 (5)	C20—C21—C12	111.7 (6)
N1—C12—C21	110.8 (5)	O1—C21—H21A	109.8
C13—C12—C21	109.2 (6)	C20—C21—H21A	109.8
N1—C12—H12A	107.5	C12—C21—H21A	109.8
C13—C12—H12A	107.5	C9—N1—C10	108.1 (5)
C21—C12—H12A	107.5	C9—N1—C12	110.6 (5)
C14—C13—C12	114.0 (5)	C10—N1—C12	108.9 (5)
C14—C13—H13A	108.7	C21—O1—H1O	108 (5)

Document origin: *publCIF* [Westrip, S. P. (2010). *J. Apply. Cryst.*, **43**, 920-925].

Table 1. Crystal data and structure refinement for 1.

Identification code	global	
Empirical formula	C22 H28 I N O	
Formula weight	449.35	
Temperature	93(2) K	
Wavelength	0.71073 Å	
Crystal system	Orthorhombic	
Space group	$P2_12_12_1$	
Unit cell dimensions	a = 9.982(2) Å	α= 90°.
	b = 13.149(3) Å	β= 90°.
	c = 14.770(3) Å	γ = 90°.
Volume	1938.6(8) $Å^3$	
Z	4	
Density (calculated)	1.540 Mg/m^3	
Absorption coefficient	1.661 mm^{-1}	
F(000)	912	
Crystal size	0.090 x 0.070 x 0.050 mm^3	
Theta range for data collection	2.07 to 25.34°.	
Index ranges	-11<=h<=12, -15<=k<=11, -16<=l<=17	
Reflections collected	9534	
Independent reflections	3524 [R(int) = 0.0595]	
Completeness to theta = 25.34°	100.0 %	
Max. and min. transmission	0.92 and 0.86	
Refinement method	Full-matrix least-squares on F^2	
Data / restraints / parameters	3524 / 162 / 221	
Goodness-of-fit on F^2	0.995	
Final R indices [I>2sigma(I)]	R1 = 0.0376, wR2 = 0.0580	
R indices (all data)	R1 = 0.0455, wR2 = 0.0612	
Absolute structure parameter	0.03(3)	
Largest diff. peak and hole	0.580 and -0.413 e.$Å^{-3}$	
