# Supplementary material for: In Vivo Differences between Two Optical Isomers of Radioiodinated o-iodo-trans-decalinvesamicol for Use as a Radioligand for the Vesicular Acetylcholine Transporter
Source: PLoS One. 2016 Jan 11;11(1):e0146719. doi: 10.1371/journal.pone.0146719 (PMC4713475; doi:10.1371/journal.pone.0146719)
Supplement: S2 File — (PDF) [file pone.0146719.s002.pdf]

Crystal Report for (+)-OIDV

Computing details

Data collection: *APEX* II ULTRA (Bruker AXS Inc.); cell refinement: *APEX2* v2013.10 (Bruker AXS); data reduction: *SAINT* v8.3C (Bruker AXS Inc.); program(s) used to refine structure: *SHELXL2013* (Sheldrick, 2013); molecular graphics: XSHELL v6.3.1 (Bruker AXS Inc.); software used to prepare material for publication: Generate Report (Bruker AXS Inc.).

(I)

Crystal data

$C_{21}H_{30}IKNO$	$F(000) = 972$
$M_r = 478.46$	$D_x = 1.551$ Mg m^{-3}
Monoclinic, $P2_1$	Cu $K\alpha$ radiation, $\lambda = 1.54178$ Å
$a = 16.7268$ (10) Å	Cell parameters from 273 reflections
$b = 7.0589$ (3) Å	$\theta = 3.7–66.7°$
$c = 17.3535$ (9) Å	$\mu = 14.15$ mm^{-1}
$\beta = 90.569$ (4)°	$T = 93$ K
$V = 2048.88$ (18) Å^3	0.08 × 0.05 × 0.04 mm
$Z = 4$	

Data collection

D8 goniometer (Bruker AXS Inc.) diffractometer	6643 independent reflections
Radiation source: rotating-anode X-ray tube, Bruker Tourbo X-ray Source	5494 reflections with $I > 2\sigma(I)$
Multilayered conforcal mirror monochromator	$R_{int} = 0.048$
ω scans	$\theta_{max} = 66.6°$, $\theta_{min} = 5.1°$
Absorption correction: multi-scan *SADABS* V2012/1 (Bruker AXS Inc.)	$h = -19 \rightarrow 19$

T_{min} = 0.51, T_{max} = 0.64	k = -8→8
14095 measured reflections	l = -20→20

Refinement

Refinement on F^2	Hydrogen site location: inferred from neighbouring sites
Least-squares matrix: full	H-atom parameters constrained
$R[F^2 > 2\sigma(F^2)]$ = 0.099	$w = 1/[\sigma^2(F_o^2) + (0.164P)^2 + 8.053P]$ where $P = (F_o^2 + 2F_c^2)/3$
$wR(F^2)$ = 0.267	$(\Delta/\sigma)_{max}$ < 0.001
S = 1.06	$\Delta\rho_{max}$ = 4.25 e Å^{-3}
6643 reflections	$\Delta\rho_{min}$ = -0.66 e Å^{-3}
451 parameters	Absolute structure: Flack x determined using 1778 quotients [(I+)-(I-)]/[(I+)+(I-)] (Parsons and Flack (2004), Acta Cryst. A60, s61).
743 restraints	Absolute structure parameter: 0.017 (8)

Special details

Geometry. All esds (except the esd in the dihedral angle between two l.s. planes) are estimated using the full covariance matrix. The cell esds are taken into account individually in the estimation of esds in distances, angles and torsion angles; correlations between esds in cell parameters are only used when they are defined by crystal symmetry. An approximate (isotropic) treatment of cell esds is used for estimating esds involving l.s. planes.

Fractional atomic coordinates and isotropic or equivalent isotropic displacement parameters (Å^2)

	x	y	z	U_{iso}*/U_{eq}
C1A	0.9101 (9)	0.254 (3)	0.1330 (9)	0.034 (3)
C2A	0.9167 (10)	0.204 (3)	0.0586 (10)	0.042 (3)
H2AA	0.9443	0.286	0.0244	0.05*
C3A	0.8841 (10)	0.037 (4)	0.0307 (9)	0.045 (3)
H3AA	0.8913	0.0005	-0.0215	0.054*
C4A	0.8430 (12)	-0.071 (3)	0.0782 (9)	0.046 (3)
H4AA	0.8182	-0.1826	0.0591	0.055*

C5A	0.8349 (11)	-0.024 (3)	0.1567 (9)	0.042 (3)
H5AA	0.8049	-0.1046	0.1894	0.05*
C6A	0.8691 (9)	0.134 (3)	0.1866 (9)	0.031 (2)
C7A	0.8667 (8)	0.181 (2)	0.2704 (8)	0.027 (2)
H7AA	0.8665	0.3222	0.2755	0.032*
C8A	0.7939 (8)	0.104 (2)	0.3127 (8)	0.025 (2)
H8AA	0.7444	0.1435	0.2854	0.03*
H8AB	0.7957	-0.0363	0.3131	0.03*
C9A	0.7925 (8)	0.177 (2)	0.3953 (8)	0.027 (3)
H9AA	0.7929	0.3174	0.3952	0.032*
H9AB	0.7432	0.1341	0.4209	0.032*
C10A	0.9398 (8)	0.166 (2)	0.3963 (8)	0.024 (2)
H10A	0.9435	0.306	0.3985	0.029*
H10B	0.9874	0.1138	0.4231	0.029*
C11A	0.9410 (8)	0.105 (2)	0.3139 (7)	0.024 (2)
H11A	0.9417	-0.035	0.3112	0.029*
H11B	0.99	0.1531	0.2892	0.029*
C12A	0.8728 (9)	0.180 (3)	0.5211 (9)	0.033 (3)
H12A	0.9113	0.2882	0.5202	0.04*
C13A	0.7960 (10)	0.250 (3)	0.5582 (9)	0.035 (3)
H13A	0.7665	0.3297	0.5204	0.042*
H13B	0.8103	0.3315	0.6027	0.042*
C14A	0.7401 (10)	0.090 (3)	0.5860 (9)	0.037 (3)
H14A	0.716	0.024	0.5404	0.045*
C15A	0.6723 (11)	0.186 (3)	0.6339 (11)	0.048 (3)
H15A	0.6962	0.2484	0.6797	0.057*
H15B	0.646	0.2851	0.6022	0.057*
C16A	0.6129 (12)	0.050 (4)	0.6585 (11)	0.057 (4)
H16A	0.5841	-0.0004	0.6128	0.068*
H16B	0.5735	0.1139	0.6917	0.068*
C17A	0.6514 (15)	-0.115 (4)	0.7031 (13)	0.061 (4)
H17A	0.6716	-0.0681	0.7534	0.073*
H17B	0.6103	-0.2125	0.7133	0.073*
C18A	0.7196 (14)	-0.204 (4)	0.6596 (12)	0.056 (4)
H18A	0.6984	-0.2678	0.6127	0.068*
H18B	0.7457	-0.3017	0.6923	0.068*

C19A	0.7827 (11)	-0.051 (3)	0.6360 (10)	0.044 (3)
H19A	0.802	0.0153	0.6837	0.053*
C20A	0.8528 (12)	-0.122 (3)	0.5936 (10)	0.049 (3)
H20A	0.8796	-0.2195	0.6258	0.059*
H42	0.8333	-0.1859	0.5462	0.059*
C21A	0.9119 (11)	0.016 (4)	0.5718 (9)	0.050 (3)
H21A	0.9549	-0.0473	0.5416	0.06*
C1B	0.5825 (9)	0.295 (3)	0.3564 (9)	0.033 (3)
C2B	0.5745 (11)	0.338 (3)	0.4353 (10)	0.039 (3)
H2BA	0.546	0.256	0.4686	0.047*
C3B	0.6090 (10)	0.501 (3)	0.4631 (8)	0.038 (3)
H3BA	0.6053	0.5309	0.5163	0.046*
C4B	0.6481 (11)	0.621 (3)	0.4156 (10)	0.044 (3)
H43	0.6706	0.7348	0.4354	0.053*
C5B	0.6554 (10)	0.578 (3)	0.3379 (9)	0.036 (3)
H5BA	0.6847	0.6606	0.3055	0.044*
C6B	0.6213 (9)	0.418 (2)	0.3069 (8)	0.029 (2)
C7B	0.6248 (8)	0.378 (2)	0.2219 (8)	0.024 (2)
H7BA	0.6275	0.2376	0.2143	0.028*
C8B	0.6972 (8)	0.469 (2)	0.1811 (7)	0.024 (2)
H8BA	0.6952	0.6086	0.1874	0.029*
H8BB	0.7475	0.423	0.205	0.029*
C9B	0.6962 (8)	0.419 (2)	0.0948 (8)	0.024 (2)
H9BA	0.7445	0.4708	0.0698	0.029*
H9BB	0.6959	0.2802	0.0881	0.029*
C10B	0.5497 (8)	0.416 (2)	0.0943 (7)	0.023 (2)
H10C	0.5505	0.2773	0.0849	0.027*
H10D	0.5006	0.4677	0.0701	0.027*
C11B	0.5478 (8)	0.453 (2)	0.1822 (7)	0.021 (2)
H11C	0.5007	0.3888	0.2045	0.026*
H11D	0.5426	0.5903	0.1917	0.026*
C12B	0.6178 (9)	0.500 (3)	-0.0306 (8)	0.030 (2)
H12B	0.5657	0.5623	-0.0433	0.037*
C13B	0.6797 (10)	0.630 (2)	-0.0663 (8)	0.031 (3)
H13C	0.6555	0.69	-0.1125	0.037*
H13D	0.6922	0.7321	-0.029	0.037*

C14B	0.7564 (9)	0.538 (3)	-0.0893 (7)	0.032 (2)
H14B	0.7864	0.5035	-0.0412	0.039*
C15B	0.8086 (12)	0.680 (3)	-0.1356 (11)	0.048 (3)
H15C	0.7787	0.724	-0.1817	0.058*
H15D	0.8215	0.7915	-0.1032	0.058*
C16B	0.8844 (13)	0.583 (4)	-0.1596 (12)	0.057 (4)
H16C	0.9158	0.5488	-0.113	0.068*
H16D	0.9168	0.6723	-0.1903	0.068*
C17B	0.8688 (12)	0.405 (3)	-0.2069 (11)	0.051 (4)
H17C	0.8407	0.4396	-0.2554	0.061*
H17D	0.9204	0.3456	-0.2205	0.061*
C18B	0.8191 (12)	0.267 (3)	-0.1626 (10)	0.045 (3)
H18C	0.8502	0.2198	-0.1178	0.053*
H18D	0.8057	0.1575	-0.1959	0.053*
C19B	0.7423 (11)	0.360 (3)	-0.1348 (9)	0.036 (3)
H19B	0.7108	0.3962	-0.1818	0.043*
C20B	0.6906 (11)	0.226 (3)	-0.0887 (9)	0.039 (3)
H41	0.6826	0.1077	-0.1187	0.046*
H20C	0.7193	0.1918	-0.0404	0.046*
C21B	0.6111 (11)	0.306 (3)	-0.0690 (10)	0.038 (3)
H21B	0.5821	0.2167	-0.0344	0.046*
I1A	0.96068 (11)	0.51202 (18)	0.16855 (8)	0.0692 (6)
I1B	0.53333 (8)	0.03311 (15)	0.31950 (7)	0.0551 (5)
K1X	0.6062 (2)	0.9248 (6)	0.0896 (2)	0.0375 (8)
K2X	0.8791 (3)	0.6716 (6)	0.4088 (2)	0.0456 (10)
N1A	0.8653 (6)	0.1031 (19)	0.4386 (6)	0.024 (2)
N1B	0.6217 (6)	0.505 (2)	0.0579 (6)	0.025 (2)
O1A	0.9448 (9)	0.096 (3)	0.6369 (8)	0.066 (4)
H1AA	0.9658	0.0113	0.6643	0.099*
O1B	0.5678 (7)	0.330 (2)	-0.1376 (7)	0.043 (3)
H1BA	0.5632	0.225	-0.1601	0.065*

Atomic displacement parameters (Å^2)

	U^{11}	U^{22}	U^{33}	U^{12}	U^{13}	U^{23}
C1A	0.030 (6)	0.040 (6)	0.033 (5)	0.006 (5)	0.001 (5)	0.001 (5)
C2A	0.035 (6)	0.057 (7)	0.034 (5)	0.006 (5)	0.010 (5)	0.003 (5)

C3A	0.044 (6)	0.061 (7)	0.029 (5)	0.003 (6)	0.000 (5)	-0.006 (6)
C4A	0.054 (7)	0.055 (7)	0.028 (5)	-0.003 (6)	0.001 (5)	-0.010 (5)
C5A	0.046 (6)	0.049 (7)	0.031 (5)	-0.005 (5)	0.006 (5)	-0.003 (5)
C6A	0.028 (5)	0.038 (5)	0.027 (5)	0.007 (5)	0.004 (4)	0.003 (4)
C7A	0.022 (5)	0.031 (5)	0.028 (4)	0.003 (5)	-0.003 (4)	0.006 (4)
C8A	0.021 (5)	0.031 (6)	0.024 (5)	0.006 (5)	0.001 (4)	0.005 (5)
C9A	0.022 (5)	0.035 (6)	0.023 (5)	0.005 (5)	0.001 (4)	0.004 (5)
C10A	0.018 (5)	0.029 (6)	0.024 (5)	0.000 (5)	-0.001 (4)	0.006 (5)
C11A	0.016 (4)	0.032 (6)	0.023 (5)	0.005 (4)	0.006 (4)	0.004 (5)
C12A	0.025 (5)	0.045 (6)	0.029 (5)	-0.005 (5)	0.000 (4)	-0.006 (5)
C13A	0.034 (5)	0.043 (6)	0.029 (5)	-0.001 (5)	0.005 (5)	-0.004 (5)
C14A	0.034 (5)	0.049 (6)	0.029 (5)	0.001 (5)	0.003 (4)	0.003 (5)
C15A	0.041 (6)	0.061 (7)	0.041 (6)	0.011 (6)	0.007 (5)	0.009 (6)
C16A	0.051 (6)	0.071 (8)	0.047 (6)	-0.001 (6)	0.011 (6)	0.005 (7)
C17A	0.065 (7)	0.066 (7)	0.051 (7)	-0.007 (7)	0.011 (6)	0.006 (6)
C18A	0.070 (7)	0.056 (7)	0.043 (6)	-0.002 (6)	0.006 (6)	0.008 (6)
C19A	0.050 (5)	0.051 (6)	0.031 (5)	0.008 (5)	0.000 (5)	0.008 (5)
C20A	0.058 (6)	0.058 (7)	0.032 (6)	0.019 (5)	0.004 (5)	0.014 (6)
C21A	0.044 (5)	0.072 (7)	0.033 (5)	0.017 (5)	-0.006 (5)	0.006 (6)
C1B	0.032 (6)	0.039 (6)	0.026 (5)	0.009 (5)	0.002 (5)	0.004 (5)
C2B	0.041 (6)	0.046 (6)	0.032 (5)	0.013 (5)	0.006 (5)	0.012 (5)
C3B	0.040 (6)	0.050 (6)	0.025 (5)	0.022 (5)	0.002 (5)	0.000 (5)
C4B	0.050 (6)	0.052 (6)	0.030 (5)	0.004 (6)	-0.003 (5)	-0.004 (5)
C5B	0.044 (6)	0.040 (6)	0.025 (5)	0.005 (5)	0.005 (5)	-0.002 (5)
C6B	0.030 (5)	0.035 (5)	0.022 (4)	0.014 (4)	0.001 (4)	0.003 (4)
C7B	0.023 (5)	0.027 (5)	0.021 (4)	0.002 (4)	0.001 (4)	0.002 (4)
C8B	0.023 (5)	0.027 (6)	0.023 (5)	0.003 (4)	0.000 (4)	0.002 (4)
C9B	0.020 (5)	0.029 (6)	0.022 (5)	0.000 (5)	-0.002 (4)	0.001 (5)
C10B	0.021 (5)	0.027 (5)	0.021 (5)	0.003 (5)	0.003 (4)	0.001 (4)
C11B	0.021 (5)	0.027 (5)	0.016 (4)	0.009 (4)	0.004 (4)	0.003 (4)
C12B	0.032 (5)	0.038 (5)	0.022 (4)	0.002 (5)	0.001 (4)	0.005 (5)
C13B	0.041 (6)	0.035 (6)	0.018 (5)	-0.005 (5)	0.005 (5)	-0.001 (5)
C14B	0.036 (5)	0.038 (5)	0.023 (4)	-0.008 (5)	0.007 (4)	-0.002 (5)
C15B	0.054 (6)	0.050 (6)	0.042 (6)	-0.017 (6)	0.019 (6)	-0.006 (6)
C16B	0.055 (6)	0.068 (7)	0.047 (6)	-0.013 (6)	0.024 (6)	-0.005 (6)
C17B	0.048 (6)	0.062 (7)	0.043 (6)	0.006 (6)	0.013 (6)	-0.004 (6)

C18B	0.051 (6)	0.048 (6)	0.034 (6)	0.012 (5)	0.002 (5)	-0.002 (5)
C19B	0.041 (5)	0.038 (5)	0.027 (5)	-0.001 (5)	0.002 (5)	0.000 (5)
C20B	0.050 (6)	0.035 (6)	0.031 (5)	-0.003 (5)	0.007 (5)	-0.003 (5)
C21B	0.042 (5)	0.039 (6)	0.033 (5)	-0.007 (5)	0.005 (5)	-0.007 (5)
I1A	0.1043 (12)	0.0392 (8)	0.0650 (8)	-0.0238 (8)	0.0490 (8)	-0.0024 (7)
I1B	0.0757 (9)	0.0405 (8)	0.0495 (6)	-0.0037 (7)	0.0296 (6)	0.0063 (6)
K1X	0.0430 (18)	0.0273 (17)	0.0424 (17)	0.0005 (15)	0.0055 (15)	-0.0032 (14)
K2X	0.054 (2)	0.0298 (19)	0.053 (2)	0.0023 (17)	0.0140 (18)	0.0002 (16)
N1A	0.015 (4)	0.038 (5)	0.020 (4)	-0.003 (4)	0.002 (3)	0.002 (4)
N1B	0.021 (4)	0.036 (5)	0.017 (4)	0.003 (4)	0.003 (3)	0.000 (4)
O1A	0.047 (6)	0.108 (9)	0.042 (5)	0.014 (6)	-0.008 (5)	-0.006 (6)
O1B	0.040 (5)	0.057 (6)	0.033 (5)	-0.009 (5)	-0.003 (4)	-0.008 (5)

Geometric parameters (Å, º) for (I)

C1A—C2A	1.34 (2)	C2B—H2BA	0.95
C1A—C6A	1.43 (2)	C3B—C4B	1.35 (3)
C1A—I1A	2.099 (18)	C3B—H3BA	0.95
C2A—C3A	1.39 (3)	C4B—C5B	1.39 (2)
C2A—H2AA	0.95	C4B—H43	0.95
C3A—C4A	1.32 (3)	C5B—C6B	1.37 (2)
C3A—H3AA	0.95	C5B—H5BA	0.95
C4A—C5A	1.41 (2)	C6B—C7B	1.505 (19)
C4A—H4AA	0.95	C7B—C8B	1.549 (19)
C5A—C6A	1.36 (3)	C7B—C11B	1.548 (18)
C5A—H5AA	0.95	C7B—H7BA	1.0
C6A—C7A	1.49 (2)	C8B—C9B	1.539 (18)
C7A—C8A	1.530 (19)	C8B—H8BA	0.99
C7A—C11A	1.543 (18)	C8B—H8BB	0.99
C7A—H7AA	1.0	C9B—N1B	1.518 (17)
C8A—C9A	1.523 (19)	C9B—H9BA	0.99
C8A—H8AA	0.99	C9B—H9BB	0.99
C8A—H8AB	0.99	C10B—N1B	1.503 (17)
C9A—N1A	1.518 (17)	C10B—C11B	1.547 (17)
C9A—H9AA	0.99	C10B—H10C	0.99
C9A—H9AB	0.99	C10B—H10D	0.99
C10A—C11A	1.494 (18)	C11B—H11C	0.99

C10A—N1A	1.519 (17)	C11B—H11D	0.99
C10A—H10A	0.99	C12B—C13B	1.52 (2)
C10A—H10B	0.99	C12B—C21B	1.52 (2)
C11A—H11A	0.99	C12B—N1B	1.538 (15)
C11A—H11B	0.99	C12B—H12B	1.0
C12A—C13A	1.53 (2)	C13B—C14B	1.49 (2)
C12A—N1A	1.534 (18)	C13B—H13C	0.99
C12A—C21A	1.59 (3)	C13B—H13D	0.99
C12A—H12A	1.0	C14B—C19B	1.50 (2)
C13A—C14A	1.55 (3)	C14B—C15B	1.56 (2)
C13A—H13A	0.99	C14B—H14B	1.0
C13A—H13B	0.99	C15B—C16B	1.50 (3)
C14A—C19A	1.50 (2)	C15B—H15C	0.99
C14A—C15A	1.57 (2)	C15B—H15D	0.99
C14A—H14A	1.0	C16B—C17B	1.52 (3)
C15A—C16A	1.45 (3)	C16B—H16C	0.99
C15A—H15A	0.99	C16B—H16D	0.99
C15A—H15B	0.99	C17B—C18B	1.50 (3)
C16A—C17A	1.54 (3)	C17B—H17C	0.99
C16A—H16A	0.99	C17B—H17D	0.99
C16A—H16B	0.99	C18B—C19B	1.52 (3)
C17A—C18A	1.51 (3)	C18B—H18C	0.99
C17A—H17A	0.99	C18B—H18D	0.99
C17A—H17B	0.99	C19B—C20B	1.52 (2)
C18A—C19A	1.57 (3)	C19B—H19B	1.0
C18A—H18A	0.99	C20B—C21B	1.49 (3)
C18A—H18B	0.99	C20B—H41	0.99
C19A—C20A	1.48 (3)	C20B—H20C	0.99
C19A—H19A	1.0	C21B—O1B	1.40 (2)
C20A—C21A	1.44 (3)	C21B—H21B	1.0
C20A—H20A	0.99	K1X—O1B^{i}	3.109 (13)
C20A—H42	0.99	K2X—N1A^{ii}	3.098 (14)
C21A—O1A	1.37 (2)	K2X—O1A^{iii}	3.103 (16)
C21A—H21A	1.0	N1A—K2X^{iv}	3.098 (14)
C1B—C6B	1.39 (2)	O1A—K2X^{v}	3.103 (16)
C1B—C2B	1.41 (2)	O1A—H1AA	0.84

C1B—I1B	2.117 (18)	O1B—K1X[vi]	3.109 (13)
C2B—C3B	1.37 (3)	O1B—H1BA	0.84

C2A—C1A—C6A	121.0 (18)	C2B—C3B—H3BA	119.6
C2A—C1A—I1A	118.1 (14)	C3B—C4B—C5B	120.2 (19)
C6A—C1A—I1A	120.9 (12)	C3B—C4B—H43	119.9
C1A—C2A—C3A	121.4 (18)	C5B—C4B—H43	119.9
C1A—C2A—H2AA	119.3	C6B—C5B—C4B	121.4 (17)
C3A—C2A—H2AA	119.3	C6B—C5B—H5BA	119.3
C4A—C3A—C2A	118.5 (16)	C4B—C5B—H5BA	119.3
C4A—C3A—H3AA	120.7	C5B—C6B—C1B	117.9 (14)
C2A—C3A—H3AA	120.7	C5B—C6B—C7B	121.2 (14)
C3A—C4A—C5A	122 (2)	C1B—C6B—C7B	120.9 (15)
C3A—C4A—H4AA	119.2	C6B—C7B—C8B	114.1 (12)
C5A—C4A—H4AA	119.2	C6B—C7B—C11B	109.4 (11)
C6A—C5A—C4A	121.2 (17)	C8B—C7B—C11B	107.8 (11)
C6A—C5A—H5AA	119.4	C6B—C7B—H7BA	108.5
C4A—C5A—H5AA	119.4	C8B—C7B—H7BA	108.5
C5A—C6A—C1A	116.1 (14)	C11B—C7B—H7BA	108.5
C5A—C6A—C7A	122.6 (15)	C9B—C8B—C7B	110.4 (11)
C1A—C6A—C7A	121.3 (16)	C9B—C8B—H8BA	109.6
C6A—C7A—C8A	114.8 (13)	C7B—C8B—H8BA	109.6
C6A—C7A—C11A	111.8 (12)	C9B—C8B—H8BB	109.6
C8A—C7A—C11A	106.3 (11)	C7B—C8B—H8BB	109.6
C6A—C7A—H7AA	107.9	H8BA—C8B—H8BB	108.1
C8A—C7A—H7AA	107.9	N1B—C9B—C8B	108.7 (11)
C11A—C7A—H7AA	107.9	N1B—C9B—H9BA	109.9
C9A—C8A—C7A	110.5 (12)	C8B—C9B—H9BA	109.9
C9A—C8A—H8AA	109.5	N1B—C9B—H9BB	109.9
C7A—C8A—H8AA	109.5	C8B—C9B—H9BB	109.9
C9A—C8A—H8AB	109.5	H9BA—C9B—H9BB	108.3
C7A—C8A—H8AB	109.5	N1B—C10B—C11B	111.6 (11)
H8AA—C8A—H8AB	108.1	N1B—C10B—H10C	109.3
N1A—C9A—C8A	109.2 (11)	C11B—C10B—H10C	109.3
N1A—C9A—H9AA	109.8	N1B—C10B—H10D	109.3
C8A—C9A—H9AA	109.8	C11B—C10B—H10D	109.3

N1A—C9A—H9AB	109.8	H10C—C10B—H10D	108.0
C8A—C9A—H9AB	109.8	C10B—C11B—C7B	110.8 (10)
H9AA—C9A—H9AB	108.3	C10B—C11B—H11C	109.5
C11A—C10A—N1A	113.3 (11)	C7B—C11B—H11C	109.5
C11A—C10A—H10A	108.9	C10B—C11B—H11D	109.5
N1A—C10A—H10A	108.9	C7B—C11B—H11D	109.5
C11A—C10A—H10B	108.9	H11C—C11B—H11D	108.1
N1A—C10A—H10B	108.9	C13B—C12B—C21B	114.3 (13)
H10A—C10A—H10B	107.7	C13B—C12B—N1B	111.8 (12)
C10A—C11A—C7A	110.5 (11)	C21B—C12B—N1B	117.3 (14)
C10A—C11A—H11A	109.5	C13B—C12B—H12B	103.8
C7A—C11A—H11A	109.5	C21B—C12B—H12B	103.8
C10A—C11A—H11B	109.5	N1B—C12B—H12B	103.8
C7A—C11A—H11B	109.5	C14B—C13B—C12B	115.9 (14)
H11A—C11A—H11B	108.1	C14B—C13B—H13C	108.3
C13A—C12A—N1A	116.6 (12)	C12B—C13B—H13C	108.3
C13A—C12A—C21A	110.3 (13)	C14B—C13B—H13D	108.3
N1A—C12A—C21A	106.8 (14)	C12B—C13B—H13D	108.3
C13A—C12A—H12A	107.6	H13C—C13B—H13D	107.4
N1A—C12A—H12A	107.6	C13B—C14B—C19B	111.9 (13)
C21A—C12A—H12A	107.6	C13B—C14B—C15B	110.3 (16)
C12A—C13A—C14A	114.1 (15)	C19B—C14B—C15B	110.6 (13)
C12A—C13A—H13A	108.7	C13B—C14B—H14B	108.0
C14A—C13A—H13A	108.7	C19B—C14B—H14B	108.0
C12A—C13A—H13B	108.7	C15B—C14B—H14B	108.0
C14A—C13A—H13B	108.7	C16B—C15B—C14B	109.3 (18)
H13A—C13A—H13B	107.6	C16B—C15B—H15C	109.8
C19A—C14A—C13A	112.4 (14)	C14B—C15B—H15C	109.8
C19A—C14A—C15A	108.8 (14)	C16B—C15B—H15D	109.8
C13A—C14A—C15A	107.0 (16)	C14B—C15B—H15D	109.8
C19A—C14A—H14A	109.5	H15C—C15B—H15D	108.3
C13A—C14A—H14A	109.5	C15B—C16B—C17B	112.6 (18)
C15A—C14A—H14A	109.5	C15B—C16B—H16C	109.1
C16A—C15A—C14A	111.8 (19)	C17B—C16B—H16C	109.1
C16A—C15A—H15A	109.3	C15B—C16B—H16D	109.1
C14A—C15A—H15A	109.3	C17B—C16B—H16D	109.1

C16A—C15A—H15B	109.3	H16C—C16B—H16D	107.8
C14A—C15A—H15B	109.3	C18B—C17B—C16B	110.7 (15)
H15A—C15A—H15B	107.9	C18B—C17B—H17C	109.5
C15A—C16A—C17A	111.5 (18)	C16B—C17B—H17C	109.5
C15A—C16A—H16A	109.3	C18B—C17B—H17D	109.5
C17A—C16A—H16A	109.3	C16B—C17B—H17D	109.5
C15A—C16A—H16B	109.3	H17C—C17B—H17D	108.1
C17A—C16A—H16B	109.3	C17B—C18B—C19B	110.9 (17)
H16A—C16A—H16B	108.0	C17B—C18B—H18C	109.5
C18A—C17A—C16A	112.2 (18)	C19B—C18B—H18C	109.5
C18A—C17A—H17A	109.2	C17B—C18B—H18D	109.5
C16A—C17A—H17A	109.2	C19B—C18B—H18D	109.5
C18A—C17A—H17B	109.2	H18C—C18B—H18D	108.0
C16A—C17A—H17B	109.2	C14B—C19B—C20B	109.5 (13)
H17A—C17A—H17B	107.9	C14B—C19B—C18B	113.5 (15)
C17A—C18A—C19A	111 (2)	C20B—C19B—C18B	112.7 (16)
C17A—C18A—H18A	109.5	C14B—C19B—H19B	106.9
C19A—C18A—H18A	109.5	C20B—C19B—H19B	106.9
C17A—C18A—H18B	109.5	C18B—C19B—H19B	106.9
C19A—C18A—H18B	109.5	C21B—C20B—C19B	113.5 (15)
H18A—C18A—H18B	108.1	C21B—C20B—H41	108.9
C20A—C19A—C14A	108.3 (14)	C19B—C20B—H41	108.9
C20A—C19A—C18A	115.6 (19)	C21B—C20B—H20C	108.9
C14A—C19A—C18A	107.0 (16)	C19B—C20B—H20C	108.9
C20A—C19A—H19A	108.6	H41—C20B—H20C	107.7
C14A—C19A—H19A	108.6	O1B—C21B—C20B	107.9 (14)
C18A—C19A—H19A	108.6	O1B—C21B—C12B	107.4 (15)
C21A—C20A—C19A	116.5 (19)	C20B—C21B—C12B	112.4 (14)
C21A—C20A—H20A	108.2	O1B—C21B—H21B	109.7
C19A—C20A—H20A	108.2	C20B—C21B—H21B	109.7
C21A—C20A—H42	108.2	C12B—C21B—H21B	109.7
C19A—C20A—H42	108.2	N1A^{ii}—K2X—O1A^{iii}	106.5 (4)
H20A—C20A—H42	107.3	C9A—N1A—C10A	108.5 (10)
O1A—C21A—C20A	109.5 (16)	C9A—N1A—C12A	113.5 (11)
O1A—C21A—C12A	108 (2)	C10A—N1A—C12A	106.7 (10)
C20A—C21A—C12A	111.0 (14)	C9A—N1A—K2X^{iv}	108.5 (9)

O1A—C21A—H21A	109.3	C10A—N1A—K2X^{iv}	98.3 (8)
C20A—C21A—H21A	109.3	C12A—N1A—K2X^{iv}	119.8 (9)
C12A—C21A—H21A	109.3	C10B—N1B—C9B	108.4 (11)
C6B—C1B—C2B	120.9 (17)	C10B—N1B—C12B	112.6 (10)
C6B—C1B—I1B	122.8 (12)	C9B—N1B—C12B	116.0 (10)
C2B—C1B—I1B	116.3 (13)	C21A—O1A—K2X^{v}	103.5 (11)
C3B—C2B—C1B	118.7 (16)	C21A—O1A—H1AA	109.5
C3B—C2B—H2BA	120.7	K2X^{v}—O1A—H1AA	82.9
C1B—C2B—H2BA	120.7	C21B—O1B—K1X^{vi}	106.0 (9)
C4B—C3B—C2B	120.9 (15)	C21B—O1B—H1BA	109.5
C4B—C3B—H3BA	119.6	K1X^{vi}—O1B—H1BA	103.4

Symmetry codes: (i) -*x*+1, *y*+1/2, -*z*; (ii) *x*, *y*+1, *z*; (iii) -*x*+2, *y*+1/2, -*z*+1; (iv) *x*, *y*-1, *z*; (v) -*x*+2, *y*-1/2, -*z*+1; (vi) -*x*+1, *y*-1/2, -*z*.

Document origin: *publCIF* [Westrip, S. P. (2010). *J. Apply. Cryst.*, **43**, 920-925].

Table 1. Crystal data and structure refinement for 1.

Identification code	global	
Empirical formula	C21 H30 I K N O	
Formula weight	478.46	
Temperature	93.0(2) K	
Wavelength	1.54178 Å	
Crystal system	Monoclinic	
Space group	P 1 21 1	
Unit cell dimensions	a = 16.7268(10) Å	α= 90°.
	b = 7.0589(3) Å	β= 90.569(4)°.
	c = 17.3535(9) Å	γ = 90°.
Volume	2048.88(18) $Å^3$	
Z	4	
Density (calculated)	1.551 Mg/m^3	
Absorption coefficient	14.151 mm^{-1}	
F(000)	972	
Crystal size	0.081 x 0.045 x 0.035 mm^3	
Theta range for data collection	5.10 to 66.60°.	
Index ranges	-19<=h<=19, -8<=k<=8, -20<=l<=20	
Reflections collected	14095	
Independent reflections	6643 [R(int) = 0.0477]	
Completeness to theta = 66.60°	99.4 %	
Absorption correction	Semi-empirical from equivalents	
Max. and min. transmission	0.64 and 0.51	
Refinement method	Full-matrix least-squares on F^2	
Data / restraints / parameters	6643 / 743 / 451	
Goodness-of-fit on F^2	1.059	
Final R indices [I>2sigma(I)]	R1 = 0.0993, wR2 = 0.2537	
R indices (all data)	R1 = 0.1131, wR2 = 0.2671	
Absolute structure parameter	0.017(8)	
Largest diff. peak and hole	4.249 and -0.664 e.$Å^{-3}$	
