# Supplementary material for: In Vivo Differences between Two Optical Isomers of Radioiodinated o-iodo-trans-decalinvesamicol for Use as a Radioligand for the Vesicular Acetylcholine Transporter
Source: PLoS One. 2016 Jan 11;11(1):e0146719. doi: 10.1371/journal.pone.0146719 (PMC4713475; doi:10.1371/journal.pone.0146719)
Supplement: S1 Table — (DOC) [file pone.0146719.s003.doc]

**S1 Table The regional biodistribution of (+)-[^3^H]-3-PPP in rat’s brain.**

| Brain Regions | Radioactivities (%ID/g) | | |
| --- | --- | --- | --- |
|  | Post-injection time | | |
|  | 15 min | 30 min | 60 min |
| Cerebral cortex | 0.67 ± 0.02 | 0.37 ± 0.04 | 0.18 ± 0.01 |
| Cerebellum | 0.53 ± 0.01 | 0.30 ± 0.03 | 0.18 ± 0.01 |

The values are the mean ± standard deviation (SD) of four rats (n = 4) at each time point.
